# Photobiomodulation and Sports: Results of a Narrative Review

**DOI:** 10.3390/life11121339

**Published:** 2021-12-03

**Authors:** Laura Marinela Ailioaie, Gerhard Litscher

**Affiliations:** 1 Department of Medical Physics, Alexandru Ioan Cuza University, 11 Carol I Boulevard, 700506 Iasi, Romania; lauraailioaie@yahoo.com; 2 Research Unit of Biomedical Engineering in Anesthesia and Intensive Care Medicine, Research Unit for Complementary and Integrative Laser Medicine, and Traditional Chinese Medicine (TCM) Research Center Graz, Medical University of Graz, Auenbruggerplatz 39, 8036 Graz, Austria

**Keywords:** photobiomodulation, sports, fatigue, low-level laser therapy, light emitting diodes, muscle damage, performance, recovery, soreness, super-pulsed lasers

## Abstract

Benefits of photobiomodulation (PBM) have been known for several decades. More recently, PBM applied in sports offers a special chance to support the modeling of the performance and recovery. Increasingly complex physical activities and fierce competition in the world of sports generate a state of psycho-emotional and physical stress that can induce chronic fatigue syndrome, failure in physical training, predisposition to muscle damage, physical and emotional exhaustion etc., for which PBM could be an excellent solution. To evaluate and identify all risk factors and the influence of PBM on health and performance in sport and for a better understanding of its effects, we did a search for “Photobiomodulation and Sports” on PubMed, to update the PBM science applied in sports, and we retained for analysis the articles published from 2014 to date. The term “PBM” is recent, and we did not include previous studies with “low level laser therapy” or “LLLT” before 2014. In the present research, PBM has been shown to have valuable protective and ergogenic effects in 25 human studies, being the key to success for high performance and recovery, facts supported also by 22 animal studies. PBM applied creatively and targeted depending on sport and size of the level of physical effort could perfectly modulate the mitochondrial activity and thus lead to remarkable improvements in performance. PBM with no conclusive results or without effects from this review (14 studies from a total of 39 on humans) was analyzed and we found the motivations of the authors from the perspective of multiple causes related to technological limitations, participants, the protocols for physical activity, the devices, techniques and PBM parameters. In the near future, dose–response experiments on physical activity should be designed and correlated with PBM dose–response studies, so that quantification of PBM parameters to allow the energy, metabolic, immune, and neuro-endocrine modulation, perfectly coupled with the level of training. There is an urgent need to continuously improve PBM devices, delivery methods, and protocols in new ingenious future sports trials. Latest innovations and nanotechnologies applied to perform intracellular signaling analysis, while examining extracellular targets, coupled with 3D and 4D sports motion analysis and other high-tech devices, can be a challenge to learn how to maximize PBM efficiency while achieving unprecedented sports performance and thus fulfilling the dream of millions of elite athletes.

## 1. Introduction

It is in the competitive human spirit to look for the best performance in both amateurs and sports professionals. In this search for incredible results and the implementation of new exercises and innovative training, a major role is played by the selection of the latest appropriate ergogenic means designed to improve physical and mental performance, endurance, but also recovery after intense muscle training.

Due to high competition, always staying on the top for professionals has become increasingly difficult due to permanent facing with the readjustments of the human body to high stress and intense training dictate by the huge tasks of physical activities. To increase muscle strength and endurance in athletes, new means of stimulating and regulating skeletal muscles are needed, in addition to improving hypertrophic and neuromuscular fitness.

An essential property of skeletal muscle is contraction, which needs energy and is achieved by sliding actin molecules (thin) on the myosin (thick) filaments, together forming the sarcomere. Myosin head also binds to adenosine triphosphate (ATP), which is the basis of the energy supply for muscle contraction. Myosin can bind to actin only when actin coupling sites are exposed to calcium ions. Tropomyosin covers the myosin-binding sites of the actin molecules, so it must be removed to uncover the binding sites on the actin, a process that also requires energy. Calcium ions will connect to troponin C molecules, modifying the pattern of the tropomyosin and obliging it to disclose the cross-bridge coupling sites on the actin. Transferring the sodium and potassium ions through the muscle membrane to keep the vital ionic gradients also needs energy, for which the ATP is the main muscle fuel.

ATP is the basic energy unit in the physiological enzymatic processes of (Na+/K + ATPase), (Ca^2^ + ATPase), and the myofilament cross-bridge cycling (myosin ATPase) in the excitable muscle cell membrane. However, the intake of ATP for muscles can last only 1–2 s. Intramuscular deposits of ATP are reduced (~5 mmol per kg wet muscle), and at a score of ATP utilization of 3.7 mmol ATP kg^−1^ s^−1^, the muscular activity could last less than 2 s if stocked ATP was the only energy source [1].

Creatine phosphate (CK), which, like ATP contains, a high-energy phosphate bond, is a rapid source of energy for ATP regeneration. CK deposits are also limited and could supply energy for muscle contractions for only 5 to 8 s. The main sources of energy for muscles remain glucose and fatty acids, whose consumption depends on the load and fitness of the subject, as well as the availability of oxygen. ATP production from cytosolic glycolysis, mitochondrial oxidation of beta fatty acids, and the citric acid cycle are tightly regulated and respond quickly to muscle demands for more ATP [2].

The amount of ATP and its supply at the right time during skeletal muscle contraction is essential both in explosive sportive events for noticeably short periods of time (seconds or minutes), for example in sprints and jumps, but also in case of long resistance efforts in which the athlete must prove endurance for hours [1,3].

Photobiomodulation (PBM), formerly known as low-power laser therapy or low-level laser therapy (LLLT), has its new terminology adopted at the joint conference of the North American Association for Light Therapy and the World Association for Laser Therapy in September 2014, with a consensus on nomenclature for *photobiomodulation* as an ideal term [4].

PBM involves the use of visible and/or infrared laser/light to biologically modulate cellular activity, to improve tissue and cell functions through the activation of cellular enzymes, so that the flux of photons is inducing several physiological changes such as: increasing ATP production, reduction in inflammation and pain, stimulation of the formation of new muscle fibers, acceleration of angiogenesis, repair, and regeneration of tissues [5,6].

PBM has been shown in several studies to be effective in cell proliferation, stimulating metabolism, reducing inflammation, and promoting tissue healing. Amidst the parameters used, the dose delivered to a certain type of tissue is crucial, because the effects will depend on it: the application of a small dose could lead to an important cellular response, but high doses can inhibit cell proliferation or even induce apoptosis. Among the most replicable consequences of PBM is the systemic decrease in inflammation, very significant for traumatic injuries or joint diseases, lung, and brain conditions [7].

Current studies on the effects of anti-inflammatory PBM at the cellular level are focused mainly on the expression of pro-inflammatory cytokines and on the migration and concentration of macrophages at the site of impact. It is known that the macrophage plays a crucial role during the inflammatory phase; the M1 phenotype has a physiological proinflammatory activity for the defense of the host in the invasion with pathogens, and the M2 phenotype participates in the remedy of injuries in the phase of extinguishing the inflammation [8].

PBM regulates through complex mechanisms a wide range of pro-inflammatory/anti-inflammatory cytokines and the level of polarization of macrophages responsible for an excessive inflammatory response or accelerated tissue healing.

Wavelength influences the propagation, flux, and rate of distribution of photons in irradiated tissues, as well as the effectiveness of non-invasive application of the laser. The wavelength used by PBM is a valuable parameter in the response to cell proliferation because the wavelength between 600–1070 nm (red/near-infrared (IR)) has the best non-invasive effects. It has been observed that the shorter wavelengths are absorbed by hemoglobin or melanin, producing cellular effects, while the longer wavelengths are absorbed by water, and give the sensation of warmth and induce pain relief [9].

From the first applications, PBM has been used for the treatment of many inflammatory diseases, musculoskeletal disorders, and especially for tissue regeneration and recovery. Intensive development of advanced laser systems—as well as other medical treatment devices—has led to the unprecedented expansion of the multitude of therapy options including stimulating and healing of muscles, tendons, ligaments, joints, etc. but also immunological conditions, the nervous system, as well as targeting the axis of the immune system—muscular system—brain, etc., and all in connection with training and physical exercises. The value of these therapies is the lack of side effects, of addiction, considered as energy methods that precisely address the energy processes inside the cells and what is most valuable, without drugs or toxic consequences.

## 2. Methodology

From previous randomized and placebo-controlled scientific LLLT studies, it is known that the wavelengths red to near-IR delivered from single laser diodes or clusters, LEDs, or arrangements of both in different impressively adaptable devices can supply energy to the cellular power plants to repair and regenerate muscles, painful joints due to intense physical activity, and restore the physiological balance. Specific muscle characteristics studied previously included parameters such as exhaustion, muscle fatigue, set of repetitions, twisting force impulse, muscle fiber hypertrophy, the degree of muscle damage, such as CK, lactate dehydrogenase (LDH), etc. and remaining muscle pain or delayed onset muscle soreness, as well as the recovery time [10].

To evaluate and identify all risk factors and the influence of PBM on health and performance in sport and for a better understanding of its effects in high elite athletes, we conducted a search for “Photobiomodulation and Sports” on PubMed, to update the PBM science applied in sports, and we retained for analysis all the articles published from 2014 to date.

The term “PBM” is recent, and we did not include previous studies with “low level laser therapy” or “LLLT” before 2014 [4].

**Figure 1:**
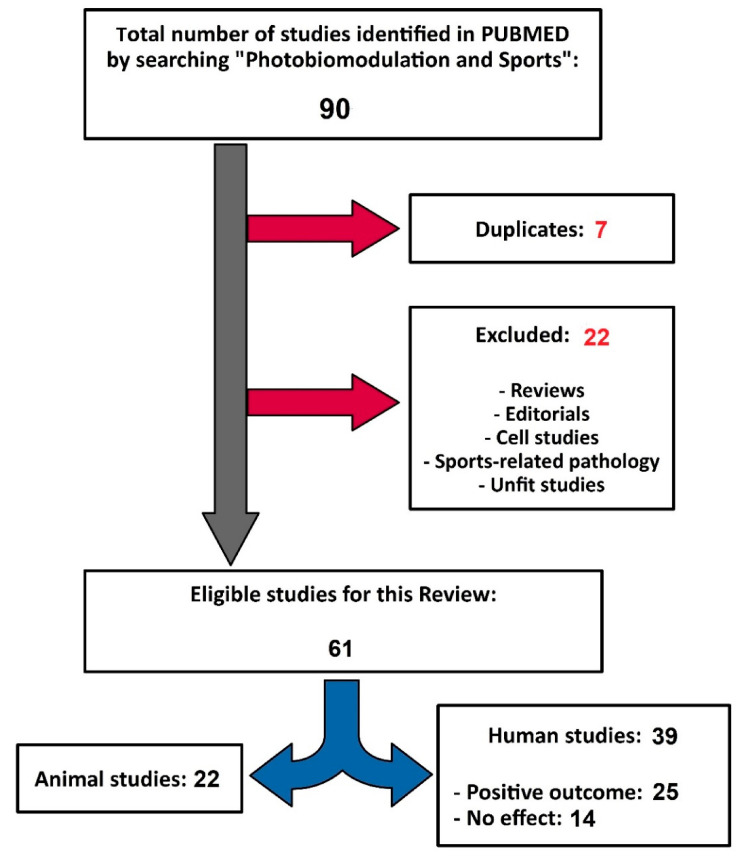
Organization chart of the studies selection.

The search retrieved 90 studies, of which 29 studies were excluded (reviews, editorials, cellular studies, sports-related pathologies, inadequate studies due to lack of randomization or control group, duplicates, etc.), and the difference (61 studies) was included in the analysis (Figure 1).

Of the last 61 studies considered in this review, 39 were in human subjects, and 22 were experimental animal studies. Analysis of research in human subjects revealed positive effects of PBM in 25 studies, which included 797 participants, and 14 studies did not indicate any relevant effect of PBM compared to control groups.

## 3. PBM Applied in Sports in Different Settings and Conditions

### 3.1. Positive Effects of PBM

Applying PBM both before and after training can have positive effects, so we classified the studies into applications of PBM before, after, before and after, and in experimental laboratory conditions when participants were running on the treadmill.

There were 39 randomized, placebo-controlled studies on humans, of which only 25 (with 797 subjects) had positive results due to PBM applied to various light physical activities or intensive training, before, after, both before and after, or under experimental laboratory conditions, of which 21 are summarized in Table 1, and four other studies with PBM and simultaneously applied static magnetic field are mentioned in the final discussions.

In order to set out the most favorable dose of PBM, Antonialli et al. [11] evaluated skeletal muscle efficiency and the recuperation after exercise including 40 vigorous male volunteers, but physically unprepared, into a randomized, double-blind, placebo-controlled study using 12 cluster diodes (4 IR laser diodes of 905 nm, 4 IR LEDs of 875 nm and, 4 red LEDs of 670 nm). They administered 10, 30, and 50 J, or placebo, in six points on the front of the thighs, using only one PBM treatment immediately after pre-exercise maximal voluntary contraction (MVC), and finally analyzing MVC, delayed-onset muscle soreness (DOMS), and the creatinine kinase (CK). Ratings were effectuated before, 1 min, 1 h, 24 h, 48 h, 72 h, and 96 h after the procedures to provoke muscular tiredness. PBM enhanced MVC from immediately after to 96 h after exercise with 10 or 30 J doses, remarkably diminished DOMS with 30 J dose from 24 h to 96 h after exercise, and with 50 J dose from immediately after to 96 h after exercise; and significantly decreased CK activity with all PBM doses, in comparison to the placebo group, concluding that the 30 J dose was the best.

In another study, Vanin et al. [12] evaluated the effects of 810 nm/200 mW PBM applied also in six sites on quadriceps with a cluster with only 5 diodes, applying 10, 30, or 50 J in a randomized, double-blind, placebo-controlled study in 28 high-level soccer athletes, also to identify the optimal dose for best recovery and performance. Researchers assessed MVC, DOMS, CK activity, IL-6 expression, before and after 1 min, 1 h, 1 day to 4 days, after the protocol to trigger the muscular exhaustion. PBM increased MVC from immediately after exercise to 24 h with 50 J dose, and from 1 day to 4 days with 10 J dose; it decreased CK and IL-6 with better results in favor of 50 J dose and had no effect on DOMS. Authors concluded that pre-exercise PBM with 50 J energy dose remarkably rose the performance and reduced the biochemical markers linked to damage and inflammation in the skeletal muscle system.

Also in athletes, but in an anaerobic field test using a randomized, crossover, double-blind, placebo-controlled clinical trial in twelve male high-level rugby players, Pinto et al. [13] demonstrated the effects of PBMT in improving performance and speeding up the rehabilitation time during the Bangsbo sprint test (BST). There were no interventions before BST in the familiarization phase (week 1) but in weeks 2 and 3, pre-exercise PBMT (at 17 points of each leg, employing a cluster with 12 diodes (4 super-pulsed IR laser diodes of 905 nm, 4 IR LEDs of 875 nm, and 4 red LEDs of 640 nm, 30 J per site) or placebo, was randomly delivered to every athlete. As a result, PBMT improved the average sprint time and fatigue index in BST and outstandingly fall down the percentage of blood lactate levels to 3, 10, 30, and 60 min after BST, initiating a novel pathway for large-scale applications of PBMT in real sports conditions.

The best PBMT output power for skeletal muscle recovery was identified by AR de Oliveira et al. [14] in a randomized, double-blind, placebo-controlled study involving 28 high-level football players. PBMT was applied before the eccentric contraction protocol with a cluster also with five diodes (810 nm, 10 J dose), but three different output powers (100, 200, 400 mW per diode) or placebo, at six sites of the knee extensors. Voluntary maximal isometric contraction (MIVC), DOMS, CK and lactate dehydrogenase, inflammation (IL-1β, IL-6, and TNF-α), and oxidative stress (catalase, superoxide dismutase, carbonylated proteins, and thiobarbituric acid) were evaluated before isokinetic exercise, as well as after 1 min and 1 h to 96 h. PBMT increased MIVC and decreased DOMS and the biochemical marker levels with the best results for 100 mW output power per diode (500 mW in total) in improving performance and post-exercise restauration.

Rossato et al. [15] aimed to identify the effects of two different time responses on knee extensor fatigue in sixteen male volunteers, distributed to perform the same protocol in 5 sessions. PBMT was applied to the knee extensor (9 sites, 30 J per site). MIVC was evaluated before and after isokinetic fatigue associated with electromyography (root mean square [RMS] and median frequency [MF]). Time effect was observed for peak torque (PT), RMS, and MF. The effect of treatment was checked for PT, and 6 h before + immediately before the condition showed higher PT during MIVC (pre to post) than control or placebo. Applying PBMT with 6 h + directly before exercises is capable to diminish tiredness.

To test PBMT effects on futsal players’ performance and recovery, De Marchi et al. [16] included six professional athletes in a randomized, triple-blinded, placebo-controlled, crossover clinical trial. PBMT was performed 40 min before matches at 17 points of every leg, also employing a cluster with 12 diodes (4 IR laser diodes of 905 nm, 4 IR LEDs of 875 nm, and 4 red LEDs of 640 nm, 30 J per site). Blood samples were collected before treatments, immediately after the matches, and 48 h after (evaluated for CK, LDH, blood lactate, and oxidative damage of lipids and proteins). Time spent by athletes on the pitch and the distance covered were video quantified. PBMT significantly increased the time of staying in the pitch and determined a meaningful improvement in all the evaluated biochemical markers, but without any statistically significant difference in the mileage. Concluding, pre-exercise PBMT can successfully increase the workout and speed up the rehabilitation process of high-level futsal players.

Because muscle tiredness is an inherent hazard for hamstring stretch lesions in soccer players, Dornelles et al. [17] investigated the effects of PBMT (300 J per thigh or placebo on the hamstrings, before the match) on twelve young male amateur soccer players in a randomized, crossover, double-blinded, placebo-controlled trial, assessed in two sessions at least at 7-day apart. Muscle endurance and useful workout were evaluated through isokinetic dynamometry and countermovement jump (CMJ) tests, respectively, before and immediately after the match. PBMT had beneficial effects on hamstring eccentric peak torque, hamstring-to-quadriceps torque ratio, and the CMJ height, respectively, compared to placebo, attenuating hamstring muscle tiredness, and thus hindering hamstring stretch injuries, which usually occur in football players.

PBM before neuromuscular electrical stimulation (NMES) is a remarkably interesting topic, investigated in a randomized, double-blind crossover trial by Jówko et al. [18] on twenty-four moderately active, healthy young men, who received 45 electrically evoked tetanic, isometric contractions of the quadriceps, preceded by PBM or placebo-PBM. The impact of PBM on muscle impairs and causes oxidative stress, as well as the return to a normal state of the muscular function after a single session of NMES, quantified by the maximal isometric voluntary muscle torques, pain, and blood samples analyzed for the muscle impairment (CK), and inflammation (C-reactive protein), were assessed from baseline to 96 h post intervention. PBM had a shielding effect on NMES-induced fall in enzymatic antioxidant protection and cut the duration of inflammation, but did not affect lipid peroxidation, muscle impairment, or restauration after NMES.

The action of pre-exercise PBMT to increase workout, speed up recuperation and attenuate the oxidative stress were examined in twenty-two male high-level soccer players treated with IR PBMT or placebo prior to a progressive running test (ergo-spirometry) until exhaustion, by Tomazoni et al. [19] in a randomized, triple-blind, placebo-controlled crossover trial (identical group). PBMT enhanced the VO2max, fatigue time, volume and time for both anaerobic and aerobic threshold appearance, and diminished CK and LDH activities, as well as TBARS, IL-6, and carbonylated protein levels; it increases SOD and CAT activities so that PBMT before exercise plays an important antioxidant effect and therefore improves athletic presentation and the post-exercise regeneration.

Da Cunha et al. [20] investigated the effects of PBM and NMES on muscle endurance, jumps’ frequency and abilities, general reactions, assessed at baseline and during follow-up at 6 and 8 weeks in a study including thirty-six volleyball athletes, randomized into three groups: control, pre-exercise PBM (IR, 850 nm, CW, 0.8 J/cm^2^, 6 J/point, total energy equal 36 J) and operational NMES on quadriceps femoris as a muscular workout (1 kHz base, 70 Hz modulation, highest intensity supportable). The greatest increase in dominant lower limb endurance was in the NMES group, as opposed to control, but for non-dominant lower limbs, the increase was present in both PBM and NMES groups (highest effect), as well as better skills to jump in the last two groups, for which muscle-stamina growth kept on for two weeks after the end of the workout, in comparison to the control.

In another study, Rossato et al. investigated the effects of PBMT applied 6 h before and immediately before exercise with a cluster of 5 IR lasers (850 nm) and 28 LEDs, as follows: 12 red LEDs (670 nm), 8 IR LEDs (880 nm) and 8 IR LEDs (950 nm) on quadricep, in a randomized, crossover, double-blind placebo-controlled study on eighteen physically active men during a complex isokinetic exercise protocol of knee extensions. It was found that exercise performance was not affected by PBMT (135 J, 270 J, or 540 J) compared with placebo, but all doses of applied PBMT led to presumptive positive effects on isometric peak torque, concentric peak torque, and concentric work compared to placebo, facilitating the same total work with less fatigue, i.e., additional sets would be possible for a higher training volume [21].

Zagatto et al. [22] have evaluated in a randomized, double-blind, placebo-controlled research, the influence of 810 nm PBM applied on the adductors directly after each physical daily workout, on inflammation, muscle impairment, and operation capacity in twenty young water polo players. Daily, before training, the physical performance was evaluated by P200 (intense swimming of 200 m) and a 30 CJ (30 s cross-jump test). Blood tests were performed for interleukins (IL) and muscle damage both before and after the physical protocol. There was no important shift in P200 in the PBMT group compared to placebo, but there was a moderate improvement in 30 CJ. IL-1β and TNF-alpha had elevated values in the PBM group at 48 h after the last treatment, compared to pre, 0 and 24 h, but did not differentiate in the two groups. IL-10 slightly increased over time in the placebo group compared to the PBM group, where creatinine kinase decreased significantly, but no important variation in lactate dehydrogenase was observed. PBM had no important effect on inflammation and muscle damage, with only a medium impact on performance. Failure of reliable results could be caused by the undersized photobiostimulation area.

PBMT and cryotherapy alone or combined for skeletal muscle rehabilitation after eccentric contractions of knee extensors were applied by de Paiva et al. [23] in 50 healthy male volunteers, randomly distributed into five groups (PBMT, cryotherapy, cryotherapy + PBMT, PMBT + cryotherapy, or placebo) for a double-blinded, placebo-controlled trial to study MVC, DOMS and the muscle damage (CK). Estimations were conducted at the starting point, immediately after, and from 1 h to 96 h, at each 24 h interval. Comparative therapies were applied 3 min after exercise and repeated every 24 h until 72 h. PBMT (905 nm super-pulsed laser and 875 and 640 nm LEDs) and cryotherapy by ice packs on pliable caoutchouc were utilized. The best for post-exercise recovery with better MVC, diminished DOMS, and CK activity from 24–96 h was singular PBMT, compared to placebo, cryotherapy, and cryotherapy + PBMT. In the PBMT + cryotherapy lot, the influence of photobiomodulation was reduced but proved important betterment in MVC, diminished DOMS and CK activity. Singular cryotherapy and cryotherapy + PBMT were comparable to placebo. Therefore, only PBMT alone could best enhance the post-physical recovery to original physiological degrees, one day after high-intensity eccentric exercises.

The efficacy of PBMT and cryotherapy, single or mixed, for muscle rehabilitation after the administration of muscular soreness exercises was one year later investigated by De Marchi et al. [24] who randomly divided forty volunteers into five groups: placebo (PG); PBMT (PBMT), cryotherapy (CG), cryotherapy-PBMT (CPG), and PBMT-cryotherapy (PCG), which underwent a protocol of four physical sessions every 24 h, measuring their MVC and testing the blood in the pre-exercise period and at 5 and 60 min post-exercise, as well as 24, 48, and 72 h later. In the first session, with a 5 min delay, it was applied 2 min PBMT and/or cryotherapy, after the MVC test. Significant increases in MVC capacity in PBMT, CPG, and PCG, compared with PG and CG, as well as a dramatic reduction in concentrations of oxidative damage biochemical markers in all muscle groups and muscular lesions (CK) in PBMT, PCG, and CPG, were registered compared with PG. PBMT really has a higher output in muscle rehabilitation than cryotherapy, which, when simultaneously applied, reduces the effectiveness of PBMT.

Recently, Vassão et al. [25] applied PBMT with a cluster consisting of 14 LEDs, as follows: 7 red diodes (630 nm) and 7 IR diodes (850 nm) on biceps brachii muscles in 32 healthy male participants randomly distributed into 3 groups: red PBM group (RPG), infrared PBM group (IPG) and control group (CG). There were analyzed the muscle fatigue using surface electromyography (EMG), blood lactate concentration, and the rate of perceived exertion (RPE) using the Borg Scale. Comparisons between groups pointed out that electromyography fatigue index decreased in the control group, but RPE and lactate concentrations increased significantly in all groups. There was no significant difference between red and infrared PBM in the reduction in muscle fatigue, but the electromyography fatigue index delta value was greater in the IPG compared with the CG, suggesting that infrared could be more effective than red in decreasing muscle fatigue.

Consecutive stimulation with PBMT (180 J) for three successive days on the bilateral femoral quadriceps with different wavelengths: infrared (IR 940 ± 10 nm), red (RED 620 ± 10 nm), mixed red and IR (RED/IR 620 + 940 nm) or placebo, on 48 male cyclists with a mean age of 33.77 years, subjected to an assessment by an incremental test, VO2max, blood lactates, exercise perception, IR detection to study heat distribution in muscles and isokinetic summing up, was performed by Carvalho et al. [26]. For 7 days long, there were accomplished reassessments 24 h from the moment of last praxis. There were no important disparities in the examined parameters under the exploratory setup. PBMT with no connection to workout was unsuccessful in improving the cyclists’ goal. Still, applying two wavelengths reveals higher success. 

Although PBM with lasers and/or LEDs on sports perfecting has been extensively investigated, not many experiments have explored the impact on strength muscular workout regarding the most favorable time for stimulation. Vanin et al. [27] randomly divided forty-eight male volunteers (18–35 years old) into four groups, who executed a robust workout, and were stimulated with PBM and/or placebo in advance, and/or after each session, using a band of probes (4 laser diodes of 905 nm, 4 IR LEDs of 875 nm, and 4 red LEDs of 640 nm). Time was 12 weeks with measurements of peak torque touched in MVC, the load in 1-RM test, and the circumference of the thigh at baseline, 4 weeks, 8 weeks, and 12 weeks. Volunteers treated with PBM before, and placebo after workout, manifested important shifts in MVC and 1-RM tests for legs, compared to other groups. Safe and without adverse effects, PBM has the capacity to rise endurance, when used before physical activities, with extra benefits in post-lesions recovery.

Felismino et al. evaluated the effects of laser irradiation on muscle injury markers after resistance exercise in a double-blind, placebo-controlled study on 22 physically active men who were randomized into two groups: laser (n = 11) and placebo (n = 11). Laser irradiation (808 nm; 100 mW; 35.7 W/cm^2^, 357.14 J/cm^2^ per point) was applied to the arms, 1 J per point for 10 s at four points of the brachial biceps of each arm, or *placebo,* between each set of biceps curl exercise. The following parameters were investigated: creatine kinase (CK) activity and maximal strength performance (1 RM) before, immediately after, 24 h, 48 h, and 72 h after the exercise-induced muscle damage protocol. Results suggested a partial attenuation of muscle injury when laser irradiation was used during exercise intervals. Maximum CK activity was attenuated after 72 h in the laser group compared to placebo, but there was no obvious positive effect on strength performance recovery [28].

De Brito Vieira et al. investigated the effects of LLLT (808 nm, 100 mW, 4 J/point), or placebo, applied to quadriceps femoris muscles between sets, and after the last series of intense exercises on fatigue resistance via the number of maximum repetitions (RM) and the electromyography fatigue index (EFI), in a randomized, double-blind, crossover trial with placebo. The participants, seven young men, clinically healthy, were allocated into two groups: active laser and placebo laser. Both groups were assessed at baseline and until the end of the study, registering the number of maximum repetitions (RM) of knee flexion-extensions in conjunction with EFI recorded by median frequency (MF). After 1 week (washout period), all volunteers were exchanged among groups, and then all assessments were repeated. LLLT increased the maximum number of RM, comparatively with the control group. For both groups, MF significantly decreased for all muscles, comparing pre- and post- evaluations at baseline and end-point. Heart rate between groups had no statistical significance. LLLT increased RM and reduced EFI, compared with the placebo group, which is helpful for high performance that demands a fast return to a normal state and less tiredness [29].

Recently, Florianovicz et al.—in a randomized controlled trial—studied the effects of two distinct PBMT protocols (red 660 nm vs. infrared 830 nm) combined with a blood flow restriction (BFR) training arrangement in wrist extensor muscles on handgrip, wrist extension force, and electromyographic comportment. Fifty-eight volunteers (clinically healthy women, aged 18–25 years old) were randomly divided into 4 groups: (1) control; (2) BFR (strengthening with blood flow restriction); (3) 660 nm + BFR; and (4) 830 nm + BFR. The hypothesis was that PBMT + BFR would increase muscle strength gain. Handgrip strength, wrist extensor muscle strength, and electromyography (EMG) of the radial carpal extensor muscle were recorded. A statistically significant increase was obtained for handgrip strength in the 660 nm group compared with the 830 nm group, and for wrist extensor strength in the 660 nm and BFR groups compared with the control group. The best increase was found for the 660 nm (red) group comparatively with the control, BFR, and the 830 nm (IR) group. Joining PBMT (660 nm) and BFR was effective for growing the handgrip strength of the wrist extensors, related to an enhancement of the electromyographic behavior [30].

Miranda et al. [31] projected in a laboratory setting, a cross-sectional study that included 20 unprepared and unexperienced male participants to receive PBMT with super-pulsed lasers combined with LEDs and to evaluate the muscle efficiency resulting from the gradual cardiopulmonary attempt on the treadmill. Subjects were administered PBMT with a 12-diode cluster in 17 points (30 J/site) on each lower limb, either with combined superpulsed lasers and LEDs, or with placebo at one session, and vice versa at the next session, and completed a cardiopulmonary test on a treadmill each time. They were evaluated for: distance traveled, time to exhaustion, and pulmonary ventilation, all three parameters that increased after effective PBMT, as well as for the dyspnea score, which decreased for real PBMT, compared to placebo.

**Figure 2:**
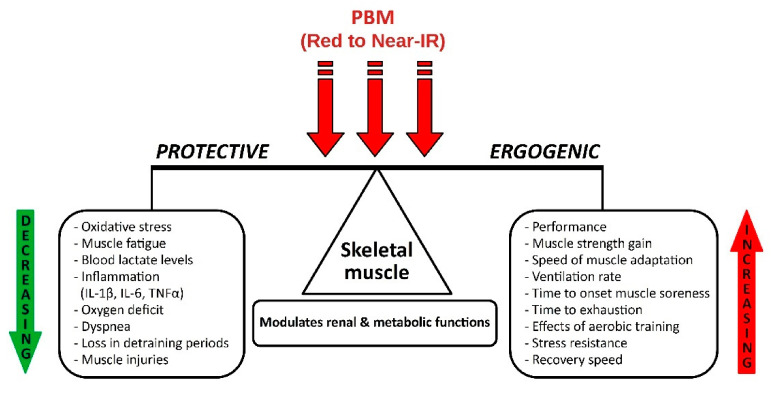
Effects of PBM on skeletal muscle in physical activity and sports.

A synthesis of the multitude of interdependent positive effects of PBM action in physical activities and sports, especially the plethora of ergogenic and protective properties, scientifically demonstrated by the positive studies analyzed, is illustrated in the original diagram designed and presented in Figure 2.

Photobiomodulation from red to near infrared had ergogenic effects by increasing performance, muscle strength, speed of muscle adaptation, ventilation rate, time to onset muscle soreness, time to exhaustion, effects of aerobic training, stress resistance, and speed of recovery.

As protective effects, PBM decreased oxidative stress, muscle fatigue, blood lactate levels, inflammation (IL-1, IL-6, TNFα), oxygen deficit, dyspnea, losses during periods without training, and muscle injuries. PBM modulates renal and metabolic functions.

### 3.2. Studies with No Effects of PBM

Fourteen studies were included in this analysis (see Figure 1).

PBMT is used today as a possible ergogenic aid for exercise performance [32] because it is believed that PBMT could have positive effects on mitochondrial enzymatic activities for ATP release [33], with beneficial effects in potential increased phosphocreatine resynthesis [34]. 

An increasing number of recently published studies have demonstrated the positive results of the acute application of PBMT on performance endurance exercises [31,35]. Although the way in which it acts and the exact mechanisms by which PBMT works successfully in increasing endurance performance in athletes are not completely elucidated, it is still considered that mitochondrial metabolism plays an essential role in this process [34,36].

PBMT through the released energy could activate the mitochondrial complex IV, increase the flow of electrons in the respiratory chain and the concentration of H+, with beneficial consequences in the high synthesis of ATP that more vigorously supports cellular activities [36] and better exercise results [37].

On the contrary, many other studies are published, some even recent, but without effects, as will be discussed below.

Dutra et al. [38] in a randomized, crossover design on 13 untrained healthy men, investigated the effects of PBMT administered 30 min or 6 h before cycling, analyzing plasma nitrogen concentrations, blood acid-base equilibrium, K+, and lactate concentrations, as well as cardiorespiratory responses were monitored during exercise. PBMT was applied to the quadriceps, hamstrings, and gastrocnemius muscles of both limbs using a multi-diode array (11 cm × 30 cm with 264 diodes) at doses of 152 J or false irradiation (with the device off, placebo). The authors demonstrated that PBMT did not improve exercise-induced changes in cardiorespiratory responses or metabolic blood markers, nor time to exhaustion during the severe intensity cycling performed by untrained men. 

Malta et al. [39] investigated the effects of PBMT on muscle recovery based on inflammation (IL-10, TNFα), markers of muscle damage (CK), LDH, DOMS, and counter-current jumping (CMJ) after two sprint interval training (SIT) sessions compared to placebo (part I) and PBMT efficacy with active recovery (AR) and cold-water immersion (CWI) [part II]. PBMT with 56 diodes 660 nm and 48 diodes 850 nm was applied at a total energy of 600 J (300 J per foot in 5 spots). In Part I, a time effect was found with increases in LDH, CK, and IL-10, as well as a decrease in DOMS to 72 h compared to 24 h. In Part II, an increase in CK and IL-10, while DOMS decreased to 48 and 72 h compared to 24 h. In summary, PBMT had no effect on inflammation, muscle injury, CMJ, or DOMS performance after two consecutive sprint interval training sessions compared to placebo, CWI, and AR strategies.

The stretching-shortening performance of the lower limb muscles during training or competition in athletes is influenced by fatigue and muscle damage. PBMT is used as a strategy to alleviate fatigue and muscle damage when applied before different types of exercises. In a placebo-controlled study, Orssatto et al. [40] investigated the effects of PBM (LASERs 850 nm, LEDs 670 nm, LEDs 880 nm, LEDs 950 nm; number of diodes = 33) applied to 15 lower limb sites with a dose per site of 30 J and a total dose of 450 J (Quadriceps = 240 J, Hamstrings = 120 J, and Gastrocnemius = 60 J). There was no effect of PBMT before exercise to reduce lower limb muscle fatigue and damage during and following a stretch-shortening cycle protocol in judo athletes. In another randomized cross-over, placebo-controlled, and double-blind design study on fourteen well-trained adults, Orssatto et al. [41] investigated the effects of PBMT on endurance training and the generated pain. PBMT (60 J per site, 6 sites per member, total dose = 360 J) was used to reduce tiredness. PBMT has not been helpful in increasing volume or reducing discomfort during resistance training and conducted to concentric failure to well-trained participants.

In another two studies (study 1 with 14 male cyclists and study 2 with 13 cyclists), pseudorandomized and balanced crossover performed by Dutra et al. [42] analyzed the ergogenic effects of two doses of PBMT (18 × 38 cm matrix with 200 diodes, at doses 260 J and 130 J or 0 J for placebo) applied before exertion, on markers of respiratory and muscular activity, blood-base acid, ion and lactate concentrations, indicators of muscle fatigue (global, central and peripheral) and time to exhaustion in the severe cycle. In conclusion, it is shown that PBMT at doses of 260 J and 130 J had no beneficial effects on muscle fatigue, cyclic performance, metabolic parameters, and muscle activity in men with recreational cycling.

Santos et al. [43] in a counterbalanced randomized crossover design (one week between PBMT/placebo (SHAM) at thirteen amateur futsal players, evaluated the effect of previous acute application of PBMT (diodes: 69; wavelength: mixed, 34 diodes 660 nm and 35 diodes 850 nm; CW, cluster area: 44.2 cm^2^; optical output: 53 mW/cm^2^; spot site area: 0.234 cm^2^; dose: 200 J; energy density: 4.5 J/cm^2^) on the performance of high and intermittent intensity exercises, muscle oxygenation, and physiological/perceptual indicators. Overall, PBMT before a battery of physical tests did not alter the performance of high and intermittent exercise, nor the physiological and perceptual responses in amateur futsal players.

As the authors claim, this research used CMJ as an essential parameter in the assessment of neuromuscular fatigue and at the same time is the first study to test the influence of PBMT in physical recovery after intense exercise performed on an ergometric cycle. Results of this research are similar to those published by Malta et al. [39] in the sense that no improvement in CMJ performance was observed after PBMT at doses of 200 and 300 J, i.e., at 4–5 J/cm^2^. Although the site chosen for local application of PBM was most appropriate in stimulating the muscle group participating in physical activity, the “ideal dose” remains the most important issue because it is not known exactly which would be most appropriate.

In many sports activities, hamstring strain injury (HSI) is the most common non-contact injury [44] and through classic rehabilitation programs [45] many athletes return to sports with a low level of performance and high risk of recurrence [46] of the disease.

Muscle injuries caused by physical activity are repaired both structurally and functionally slowly. Experimental animal studies have tested the positive biochemical and histological effects of PBM [47,48]. The use of PBM in experimental models began immediately, i.e., in the first 24 h after muscle injury, the time essential for intervention on the inflammatory process [49]. This hypothesis could explain the inefficiency of PBMT reported by Medeiros et al. [50] following a randomized controlled study with 24 amateur athletes who received adjunct therapy PMB (859 nm, 100 mW, CW, 5 diodes, 30 J site for 3 sites) for functional rehabilitation of the HSI produced through an intense program of physical exercises, 48 to 96 h ago. As the authors point out, the published study has limitations: first, the two-arm design prevented them from identifying whether other doses of PBM would have had different effects on the results; second, no patient-reported outcome measures were used, and ultimately, the number of participants did not constitute a large-scale study.

Mechanisms by which PBM regulates ergogenic activity are supported by the fact that radiation emitted in the red and near-infrared is absorbed into the cell by subcellular chromophores in the mitochondria [51]. Then, the enzyme cytochrome C oxidase is activated, which stimulates the production of ATP in the mitochondrial respiratory chain and thus will stimulate fiber maturation and muscle repair through increased synthesis of myoregulatory factors such as myogenin and MRF4 [52,53]. As already shown in the study published by de Oliveira et al. [14], PBM can induce muscle protection in athletes through the action of reducing inflammation and CK levels [54] produced during physical activity.

Results of the PBMT depend on many factors, among which we mention the parameters of the devices to work with: wavelength, fluency, illumination time, spot size, and the method of delivery (e.g., contact, punctual, wide beam). Among the mentioned PBM parameters, the ones that matter the most are the estimated power density in mW/cm^2^, and the fluence expressed in J/cm^2^ [55].

Ghigiarelli et al. [56] included twelve men in a randomized counterbalanced crossover project, and examined the effects of PBMT (mixed, 660 nm and 850 nm, with 2800 diodes, total energy emitted in 15 min 473 J, respectively 400 J) administered for 15 min immediately before and after high-intensity resistance training. They measured CK and IL-6 levels. Finally, PBMT did not significantly reduce the activity of salivary IL-6 or CK concentration during post-intensity recovery endurance training for 24 to 72 h. These negative results could be explained, according to the authors, by not using the recommended doses of 60–300 J for large muscle groups and 20–60 J for small muscles suggested by Leal-Junior et al. [57].

PBM has begun to be used in athletes as a recent way to prevent muscle injury and pain after strenuous physical activity [58,59]. As already mentioned, this treatment is scientifically based on the assumption that PBM stimulates mitochondrial chains and the cytochrome C oxidase enzyme, which increases ATP production and thus delays muscle fatigue and protects muscle from injury [60,61]. PBM stops the release of muscle damage markers (LDH and CK), proinflammatory protein production (CRP), improves the oxidative system, and increases muscle plugging power [62,63]. Studies on the effects of PBM on muscle performance and fatigue, with various protocols related to wavelength, power density, irradiation time, and laser light sources still reported discordant results [64,65].

In a double-blind, placebo-controlled randomized clinical trial, Yekta et al. [65] investigated the effect of PBM on muscle strength and endurance and post-exercise recovery of 50 young adults aged 20 to 35 years. The first group received pre-exercise laser at 810 nm, 60 mW, and 60 Hz frequency for 30 s on the three-point rectus femoris muscle. The influence of PBM on lactate level, weight repetition, fatigue, and muscle pain in both the treatment group and the placebo group was studied. The authors conclude that taking PBM before exercise can improve muscle performance and reduce muscle pain and fatigue.

In a double-blind crossover design study, placebo-controlled with 15 moderately active and healthy males, Malta et al. [66] investigated the acute effects of PBMN using cluster light-emitting diodes (LEDT; 104 diodes) (wavelength 660 and 850 nm; energy density 1.5 and 4.5 J/cm^2^; energy 60 J at each point; total energy delivered 600 J) on the maximal deficit of oxygen (MAODALT) and time to exhaustion, during a high-intensity running effort. No significant differences were found for the values of oxygen equivalents from each energetic metabolism between PBMT subjects and placebo, or for the time to exhaustion, except for the respiratory exchange ratio. PBMT after a high-intensity running effort did not alter the MAODALT, metabolic energy pathways, or high-intensity running performance.

Peserico et al. [67] investigated the effects of PBM on endurance training in thirty untrained individuals in a randomized, placebo-controlled study. PBMT was administered in 5-foot locations, with a dose of 60 J at each point and a total energy delivered per foot of 300 J, using LED equipment with 56 red light (660 nm) and 48 infrared (850 nm). The authors claim that the results of the research parameters in the two groups did not vary significantly, but in sports, a small boost can separate the winner from the loser. The inferential analysis did not show clear significant differences in Vpeak and t5-km for the PBM group compared with placebo, and only a moderate effect in relieving muscle pain in the third week of training.

In another randomized, parallel, and double-blind study in 13 young male athletes, Zagatto et al. applied whole-body photobiomodulation therapy or placebo after official water polo matches and investigated the markers of inflammation, hormonal responses (testosterone and cortisol), heart rate variability, maximal voluntary contraction, squat jump, and the muscular damage. Whole-body PBMT had no effect in improving hormonal and cardiac responses or recovery from the inflammatory muscle damage, except a decrease in LDH, but only after one match [68].

Segabinazi Peserico et al. investigated in 15 physically active males in a randomized, crossover, double-blind, and placebo-controlled study, the acute effects of PBM [56 diodes of red light (660 nm; 50 mW/cm^2^ and 1.5 J/cm^2^ each diode) and 48 diodes of infrared light (850 nm; 150 mW/cm^2^ and 4.5 J/cm^2^ each diode)] using different doses, as follows: 30 J per area (PBM1), 120 J per area (PBM2), and 180 J per area (PBM3) applied in two regions of the quadriceps muscle, two regions of the femoral biceps muscle, and one region of the gastrocnemius muscle in both legs, performed 5 min before the maximal incremental treadmill tests for the determination of peak running velocity (Vpeak) and other physiological parameters. Various doses of PBMT did not alter Vpeak and other physiological and perceptual parameters in any of the four groups [69].

Dellagrana et al. studied the running performance at 1500 m and the individual response capabilities of nineteen recreationally trained runners in a randomized, crossover, double-blind, placebo-controlled trial, applying PBMT (or placebo) 30 J per site, with a total energy dose of 840 J, in 14 sites per lower limb immediately before the stopwatch test, using a mixed-wavelength device [33 diodes: 5 LASERs (850 nm), 12 LEDs (670 nm), 8 LEDs (880 nm), and 8 LEDs (950 nm)]. In this experiment, PBMT applied immediately before running did not improve the performance of runners at 1500 m, however, 10 participants had only a higher aerobic capacity than those who did not respond at all [70].

Moreover, Abreu et al. included 30 healthy, physically active young men in a randomized, double-blind, placebo-controlled, crossover study with two equal arms: PBMT (60 J; 1152 mW; 52 s; and 166.75 cm^2^) applied to brachial biceps through a flexible series of LEDs and placebo, investigating the time response (5 min, 1 h, 3 h, and 6 h) of PBMT on maximum torque (PT), torque development rate (RTD), fatigue strength and subjective perception of effort in maximal voluntary isometric contractions (MVIC) of elbow flexion. PBMT by LEDs was performed using a flexible array of 132 LEDs, as follows: 60 red LEDs (635 ± 10 nm; 1.2 mW), 72 IR LEDs (880 ± 20 nm; 15 mW), with 4.7 kHz emission frequency and an effective irradiation surface of 166.75 cm^2^. PBMT failed to increase muscle performance, nor did it reduce fatigue [71].

Regarding the influence of PBM on the chronic effects of endurance running training, research is needed to analyze the result on biomarkers related to muscle pain, inflammation, and oxidative metabolism, depending on the doses administered.

A summary of the no-effects studies included in this review, discussed above, is presented in Table 2.

## 4. Discussions

PBM applied in sports offers a special chance to support the modeling of the performance both of amateurs and athletes for better training and, also, for excellent results in high elite competitions.

Increasingly complex physical activities and fierce competition in the world of sports generate a state of psycho-emotional and physical stress that can induce chronic fatigue syndrome, failure in physical training, predisposition to muscle and other tissue damage, low capacity, physical and emotional exhaustion, and the setup of different other diseases.

The biggest questions about PBM applied in sports are still open, as follows [10]:

- Optimal wavelengths, optimal time, before or after, or both, and at what interval of physical activity?
- Optimal PBM parameters (power density, fluency, modulation frequency)?
- The number of points for each muscle?
- The interaction of PBM with muscles and the chain of biochemical reactions triggered inside cells and ultimately reflected in increased performance in sports?
- Considering the notorious biphasic dose response, typical of PBM and its interaction with muscles, i.e., could it be managed, controlled or achieved without great difficulty to apply exactly as much energy as we need and not too much irradiation?
- Is it right to combine different light sources, i.e., both lasers and LEDs?

Optical profile (or focus on different deep tissues) affects the power density in tissue and should be addressed in clinical trials, as it may influence the effects of PBM.

PBM in sports, even it is a controversial subject, has real effects as a non-invasive therapy that works at different levels in the human body, causing chemical reactions of light in cells, tissues, treated organs, and even throughout the body, via the immune system, nervous system, etc.

However, so far, the use of PBM in increasing performance and athletic recovery is mainly affected by inconsistency in research, lack of standardized protocols, comparing the results of studies conducted on amateurs, with others on elite athletes, but also on different sports, etc., as we found in this review.

For recovery and for achieving high levels of performance, specific metabolic needs of the athlete must be met, dictated by the nature of the practiced sport.

During sports, intense muscle mobilization causes the release of chemical mediators with pro-inflammatory activity, by increasing the level of lactate and creatine kinase in the blood, which will cause pain, muscle fatigue, local hypoxia, change in electrolytes and membrane potentials, with consequences in the muscular recovery and the subsequent performance of the athlete [72]. With respect to the decrease in the chemical mediators in the blood (lactate and CK), PBM is effective when applied pre-exercise or post-exercise, but the lowest level of these blood parameters was found in some studies when is applied post-exercise [12,73,74].

Of all the means, as we found in the studies analyzed in this review, the best protective and ergogenic effects were obtained by using high-performance PBM applied with doses between 10–60 J, especially before exercising.

Very recent research has studied the immediate effects of exercise on antioxidant proteins, such as Nrf2 (nuclear factor erythroid 2-related factor 2), naturally present in living organisms, with a safeguarding function against oxidative stress, whose number grew very quickly after several tens of seconds of physical activity [75], and future research is also aimed at long-term reactions, i.e., such chronic reactions to training.

Nrf2 activity multiplicates with physical activity and is necessary for the basic activity of mitochondria and for the synthesis of specific mitochondrial proteins after training, providing cellular defense by upregulation of enzymes that inhibit the production and accumulation of ROS in striated muscles [76].

Nrf2 is a key regulator of the endogenous defense system in cells, ruling the expression of hundreds of cytoprotective proteins, in addition to many antioxidant enzymes [77].

Experimental animal research (Table 3) supports the beneficial effects of PBM applied before and after exercise in physical activity, through improved performance in endurance training [78,79], decreased level of proinflammatory cytokines [80,81,82,83,84], positive effects on aerobic metabolism [85,86], oxidative stress [86,87,88] and increased ATP [89,90]. Histopathological examination performed after PBM showed that it can protect myonecrosis, reduce pro-inflammatory cell infiltration, muscle destruction, inflammation and can help accelerate tissue repair [91,92,93].

Six other animal studies with positive effects of PBM and improved muscular function in a dose- and wavelength-dependent manner, the last of which with a simultaneously applied static magnetic field, are presented below [94,95,96,97,98,99].

In an experiment conducted by Leal-Junior et al. to evaluate the effects of preventive treatment with superpulsed LLLT (904 nm, 15 mW, 700 Hz, 1 J) or placebo-LLLT at one point overlying the tibialis anterior muscle (bilaterally), 5 times per week for 14 weeks on the progression of dystrophy in ten *mdx* (with a point mutation within the dystrophin gene) mice, randomly divided into two experimental groups, studying the morphological changes, creatine kinase (CK) activity, and mRNA gene expression, proved to decrease morphological changes, skeletal muscle damage, and inflammation, therefore, LLLT has potential to decrease the progression of Duchenne muscular dystrophy [94].

A subsequent study by Silva et al. showed that *mdx* mice treated with LLLT (808 nm, 30 mW output power, 1071 W/cm^2^ power density, 3 J/point, 100 s) at a single point of gastrocnemius muscle of the hind paw showed significantly lower levels of creatine kinase and oxidative stress than controls, when subjected to high-intensity forced exercise on an electric treadmill, had a beneficial role on the performance of skeletal muscle, but LLLT could not change the morphology of dystrophic muscles [95].

In a very recent study, Tomazoni et al. investigated comparatively the influence of PBMT and anti-inflammatory drugs following a diversified protocol in *mdx* mice. Finally, the researchers concluded that only PBMT treatments in combination with corticosteroids or nonsteroidal anti-inflammatory drugs gave promising and optimistic results in the skeletal muscle of *mdx* mice. Therefore, it opens up a possible future alternative in the treatment of Duchenne muscular dystrophy [96].

In an investigation conducted by Santos et al. which aimed to evaluate the effects of PBM immediately before tetanic contractions in the development of skeletal muscle fatigue and possible tissue damage in rats, which received 1, 3, or 10 J with three lengths of different wavelengths (660, 830 and 905 nm) before six tetanus contractions induced by electrical stimulation, it was shown that the doses of 1–3 J/660 nm and 1, 3 and 10 J/905 nm provided the best results, protecting skeletal muscle tissue against damage, i.e., the combined use of wavelengths can have therapeutic advantages [97].

A study by Albuquerque-Pontes et al. by applying LLLT before exercise with different doses (1, 3, and 10 J) and distinct wavelengths (660, 830, and 905 nm) with a power of 50 mW on healthy intact muscles in rats, showed an increase in cytochrome c oxidase expression and the delayed onset of fatigue, in a dose- and wavelength-dependent manner. The authors believe that the simultaneous combined use of different wavelengths can increase the performance of skeletal muscle in different conditions [98].

In another study, Albuquerque-Pontes et al. demonstrated that PBMT and a simultaneously applied 35 mT static magnetic field had positive effects, stopping the degradation of skeletal muscles and delaying the progression of Duchenne muscular dystrophy and thus paving the way for new possibilities for the non-pharmacological treatment of this genetic disease by modulating dystrophin gene and protein expression [99].

Over the last decade, the ergogenic effects of PBMT performed with laser diodes or LEDs have been shown to increase athletic performance and speed up recovery after training. Yet, many of these repercussions, as well as the complex clinical applications are still incompletely known. Some positive effects were also obtained by simultaneously applying PBMT and static magnetic fields, which we considered in this review [100,101,102,103].

It is not well known how the magnetic field acts at the subcellular level simultaneously with PBM, which directly modulates mitochondrial activity. Indeed, mitochondria stimulated by PBM play an important role in muscle recovery but concerning simultaneously static magnetic field exposure we do not know to date the exact role it plays in living cells. Future and more standardized studies are required for a better understanding of the molecular mechanisms underlying PBM and magnetic field potential challenges in order to improve research strategies.

Some studies have tried to use devices with high-power laser/light and short irradiation time. To date, high-power laser/light research is rare, the effects are not yet fully understood, and it is possible that the high-power laser/light in a very short time will cause unforeseen muscle damage to elite sportsmen (estimated at USD millions), on long-term. In a study by De Marchi et al., the low-power pulsed laser/light showed better results than the low-power continuous laser/light or the high-power continuous laser/light on a group of forty untrained healthy male participants. It is possible that muscle cells and subcellular organelles need time to organize and use a large amount of received photoenergy and cannot receive as much energy in a very short time, but only quantified. This study clearly demonstrated that high-power continuous laser/light had no effect on improving performance or post-exercise recovery [104].

Application of PBM lacking conclusive positive results or without effects in the analyzed studies was discussed in each paper by the authors (the majority of these studies was published by the same research group) and the causes were multiple, among which we mention:

- Most authors consider that the optimal parameters of PBMT (wavelength, output power, energy density, energy per stimulated point, total energy delivered, the optical profile or the shape of the beam emitted by the probe, etc.) are not strictly stipulated, but only in intervals of acceptance or recommendations.
- Another important influencing factor was the multitude of devices for PBM applications in sports (cluster, single or multiple diodes, etc.), from which researchers could choose.
- In some studies, researchers have failed to measure vessel diameter and local microcirculation in order to draw an objective conclusion on the impact of PBM.
- The effectiveness of PBM in improving sports performance is supported by some studies, but not all findings are consistent, as the PBM doses used in simple joint tests may not be sufficient for other complex sports, for example, cycling. Doses of PBM and the exercises adopted in some studies did not correspond to the complexity of the sport investigated, in order to increase the performance.
- The motivation for the lack of PBM effects in some studies was related to the choice of the study group as training level (amateur or elite), differences in adaptive muscle structure and cellular bioenergetics, age, sex, skin color, subcutaneous adipose tissue structure, etc.
- Other factors involved in the results obtained were the time (before/after, or both) chosen for PBM stimulation in relation to the exercises protocol, the evaluation times of PBM effects but also of physiological parameters, well-controlled recovery methods between two successive tests (for example, the quantity and quality of sleep between two experimental sessions), accumulation of fatigue from one exercise test to the next, etc.
- Some studies did not perform histopathological muscle examinations, and others did not perform all the blood collections necessary to draw relevant conclusions at the right time.

In terms of results carried out with whole-body PBM devices, these did not produce positive outcomes, as reported by Ghigiarelli et al. [56], or other researchers in a very recent study [68].

The underlying mechanisms of muscle injury are not universal due to inter-subject variability and training programs, and the previous muscle damage, the so-called “repeat bout effect” provides resistance to subsequent damage, therefore, in their experiment, Ghigiarelli et al. found the presence of the above-mentioned effect from week 1 to week 2, which may have attenuated the possible positive effects of PBMT. Meanwhile, the control treatment for different parts of the body was not possible because PBMT was performed with a bed, applied to the whole body and not to the limbs individually. In the future, the authors suggest the implementation of extended washing periods of at least 4 weeks or the use of parallel groups. Limitations to be considered in this study were a great between-subject variability post-exercise for salivary IL-6, lack of blinding of PBMT to the participants and primary investigator, absence of direct contact with the skin/target tissue of the PBM whole-body device, and the presence of the repeat bout effect [56].

Therefore, we consider that whole-body PBMT has as its main limitation the lack of contact with the target tissue, and the optical profile (or focus on different deep tissue) affects substantially the power density in the muscles and the modulation of the mitochondrial activity, and so the effects of the whole clinical trial are corrupted.

In addition, in a series of three studies recently published in 2020 in the same volume and issue of the journal *Photobiomodul Photomed Laser Surg.*, the results of PBMT were negative [69,70,71], although a study with positive effects was published here, too [30].

There is an urgent need to improve PBM devices and delivery methods. Recently, Hanna et al. [105] demonstrated an improved cell growth and differentiation of the osteoblasts with treatments using a flat-tipped hand-piece profile in comparison with the standard Gaussian probes, and that the treatment power varied greatly by moving the standard gaussian probe from the position in contact, at several centimeters away. The huge therapeutic potential of PBM has been very recently reaffirmed in a new article published by Amaroli et al. The authors designed a novel hand-piece with a flat-top beam profile of irradiation and they compared it with other classical beam profiles, investigating the effects on mitochondrial activity (by assessing ATP synthesis) and the mitochondrial damage. The uniform distribution of the power density with this new flat-top prototype increased homogenously the production of ATP, both in the center and the edges of the beam, so using an optimum optical profile can improve the rigor and reproducibility of PBM clinical outcomes [106].

Recently, Leal-Junior et al. published some current clinical and scientific recommendations and future directions for the application of PBMT in sports. In this masterclass article, the authors, as pioneers in the field, analyzed the exponential progress made over a decade since the publication of the first randomized controlled trial on improving athletic performance and accelerating post-exercise recovery by applying PBMT to date, based on the most obvious scientific evidence with meta-analysis and randomized controlled trials. All the guidance and directions presented are only for youth/adult healthy participants, using lasers and/or LEDs. It is recommended that devices for the application of PBMT have wavelengths between 640 nm–950 nm, more recently combined simultaneously red and infrared, pulsed or continuous mode, with a power of 50–200 mW per diode (individual probes) and 10–35 mW per diode (for cluster probes), without caloric effects, in direct contact with the tegument with a slight pressure. The doses that should be applied are 20–60 J for small muscle groups and 60–300 J for large ones, at least 30 s per point, from 5 min to 6 h before physical exercises. The authors recommend that all future experiments comply with this guide [57].

In a Guest Editorial, Ferraresi C. made valuable comments on the action of PBMT with lasers and/or LEDs in sports, starting on the historical vein with the first concrete examples that had no obvious positive effects, followed a decade by two other representative studies on rats, which opened the premises of the two main modalities of applying photobiomodulation today on skeletal muscle, before and after physical exercise. The author mentions that the first two randomized clinical trials (RCTs) published simultaneously in 2008, were followed by numerous other randomized clinical trials or in vivo laboratory studies that have shown as following main effects of PBMT improved exhaustion resistance, higher peak torque, or maximum force, and prophylaxis of muscle injuries. In addition to the mechanisms of action of PBMT being highly introspected, two other essential aspects have been intensively researched: the beneficial therapeutic window, i.e., the dose–response, and the temporal physiological response in case of prior application of PBMT to physical exercises. Based on updated publications, the energy therapeutic windows for acute responses are 20–80 J (small muscle groups) or 56–315 J (large) and 18–240 J in chronic responses; and a time window of 3 to 6 h for preconditioning. Contradictory results of the published PBMT efficiency are based more only on the analysis of the applied energy (J), but not on the way of supplying this energy, i.e., high or low power (W) in a short or longer time (s), which depend on the characteristics of so many devices applied in sports (laser probes, LED probes, group of LEDs or plus laser diodes, matrices displayed in the form of blankets, LEDs plus laser diodes combined with magnetic field therapy and, more recently, light beds for full-body PBM), with different irradiation areas and especially, diverse energy densities [107].

Published papers analyzed in this review reveal the important link between the applied dose, but especially its deep penetration into the tissue (optical profile), which remains an open problem to be optimized and applied creatively in sports reality, which covers as an umbrella of different sports, the diverse exercise protocols, various subjects involved in the research (athletes or amateurs), trained or untrained, different in age, sex, healthy, or with various diseases.

A permanent supply of muscle cells with ATP during sports activities modulates sports performance from very short times (seconds) to several hours, so it is necessary to activate other metabolic pathways, “anaerobic” and “aerobic” to resynthesize ATP so necessary, adapted to the intensity and duration of exercise. PBM has been shown in some studies to interfere with muscle metabolism with ergogenic benefits in sports activities. However, scientific approaches to whole-body exercise metabolism are either physiological or, more recently, through intricate analysis of cell signaling and molecular changes, which use integrated elements of genomics, proteomics, metabolomics, and systems biology in complex statistical evaluations to reveal molecular aspects of the exercise metabolism. Many questions remain open about optimal training interventions and the potential for manipulating metabolic responses, for example, through PBM, for ergogenic benefits and athlete success [1,3].

The effectiveness of PBM is not always easy to demonstrate and comprehend because its effects are present but may occur later and are not always obviously expressed. Understanding the mechanisms of action of PBM at the molecular and cellular level will allow researchers to more accurately establish optimal parameters for benefits in sports [108]. These are based on the processes of mitochondrial fission and fusion that are permanently dynamic and make the mitochondria lastingly adapt their function to the cellular requirements [109].

The best athletes bring meaningful information about their own high health costs that they “pay” in competitions or intense physical training. Regular exercise is beneficial, but strenuous competition and extreme physical training sessions could lead to significant metabolic disorders. Extremely intense exercises affect mitochondria, the cellular bioenergetic powerhouses, and decrease the body’s ability to regulate various physiological parameters in the blood. Mitochondrial impairment is associated with other dysfunctionalities [110].

Flockhart et al. studied the link between the muscular effort during physical training and the body’s response in healthy subjects, which satisfies a dose–response connection. There is a metabolic disadvantage of excessive exercise because it affects the mitochondria and reduces the body’s ability to regulate carbohydrates in the blood. Sports performance improves as the intensity of training increases, and at the same time there is an acceleration of energy production by mitochondria; at a certain level of physical effort, there is stagnation and even a decrease in performance, simultaneously with the disruption of mitochondrial activity, which decreases drastically in strong workouts, aspects proven on muscle biopsies. The research was performed on both professional athletes during endurance training but also on amateur, non-athletes. This reduction in mitochondrial activity coincided with affecting the body’s ability to metabolize glucose in the blood. Professionals showed a higher level of carbohydrates in the blood over a longer period than amateurs did. In conclusion, extreme training results in decreased performance, it induces significant mitochondrial respiratory breakdown, which is well correlated with low glucose tolerance, but despite all this, the parameters of global oxidative stress remained unchanged [110].

Multiple differences between the devices with which PBMT can be applied, constantly growing through new inventions in laser/LEDs technology and the manufacturing industry, the total amount of laser/light energy that can be used in therapy, the energy density applied to muscles, and more recently the importance of the optimum optical profile (or focus on different deep tissues) that affects the power density in tissue, and especially the mitochondrial activity, the ATP synthesis, and the mitochondrial repair, should be addressed dedicated in the future clinical trials.

Uniform distribution of the power density with the newest flat-top probes could increase homogenously the production of ATP within each cell, so using an optimum optical profile for rigorous contact and quantum-controlled muscle photobiomodulation, could improve in the near future the rigor and the reproducibility of clinical outcomes.

Many other scientific studies are still needed to elucidate all the real effects of PBM in sports, which could benefit from the unprecedented technological evolution in recent years, and which would reveal new aspects of the influence of PBM from the molecular, to the whole-body level.

## 5. Conclusions

Of all physical factors applied for the modulation of sports activities and rehabilitation, PBM has the most valuable proven protective and ergogenic effects, being the key to success for high performance and recovery.

PBMT with no conclusive results or without effects from this review was analyzed and we found the motivations of the authors from the perspective of multiple causes related to technological limitations, participants, the protocols for physical activity, the devices, techniques, and PBM parameters.

It is possible in a short time that PBM applied creatively and targeted depending on the sport and the size of the level of physical effort to perfectly modulate the mitochondrial activity and thus lead to remarkable improvements in performance, coupled with the molecular energy processes in muscle cells and their energetic inner powerplants, the regenerative mitochondria.

In the near future, dose–response experiments on physical activity should be inspired and correlated with PBM dose-response studies, so that quantification of PBM parameters to allow the energy, metabolic, immune, and neuro-endocrine modulation, perfectly coupled with the level of training.

There is an urgent need to continuously improve PBM devices, delivery methods, and protocols in new ingenious future sports trials.

Uniform distribution of the power density with the newest flat-top probes could increase homogenously the production of ATP within each cell, so using an optimum optical profile for rigorous contact and quantum-controlled muscle photobiomodulation, could quickly improve the rigor and the reproducibility of clinical outcomes.

Latest innovations and nanotechnologies applied to perform intracellular signaling analysis, while examining extracellular targets, coupled with 3D and 4D sports motion analysis and other high-tech devices, can be a challenge to learn how to maximize PBM efficiency while achieving unprecedented sports performance and thus fulfilling the dream of millions of elite athletes.

## Figures and Tables

**Table 1:** Positive effects of PBM in physical activities.

No	References	Type of Study	Type of Light/Devices for Biostimulation	PBM Before/After Activity	Trial Protocol	PBM Characteristics	Total Energy (J) Applied	Types of Physical Activities	Stimulated Muscles	Time of Assessments	Analyzed Parameters	Brief Results
				**Before**								
[11]	Antonialli, F.C.; De Marchi, T.; Tomazoni, S.S.; et al. Phototherapy in skeletal muscle performance and recovery after exercise: Effect of combination of super-pulsed laser and light-emitting diodes. *Lasers Med. Sci.* **2014**, *29*, 6, 1967–1976. doi:10.1007/s10103-014-1611-7.	RCT	Laser +LEDs	Pre-exercise PBM	Forty male healthy untrained volunteers	Twelve diodes (4 of 905 nm lasers = 0.3125 mW/each), 4 of 875 nm LEDs = 17.5 mW/each, and 4 of 670 nm LEDs = 15 mW/each.	10 J, 30 J, and 50 J or placebo	Eccentric exercise protocol to induce fatigue	Six sites on quadriceps	Before, 1 min, 1 h, 24 h, 48 h, 72 h, and 96 h	MVC DOMS CK	MVC ↑ (*p* < 0.05) immediately after to 96 h (best results) with 30 J dose. DOMS ↓ (*p* < 0.05) with 30 J dose from 24 h to 96 h after exercise DOMS ↓ (*p* < 0.05) with 50 J dose from immediately after to 96 h after exercise CK ↓ (*p* < 0.05) compared to placebo with all doses from 1 h to 96 h after exercise (except for 50 J dose at 96 h). Conclusion: Pre-exercise PBM (combined laser and LEDs), mainly with 30 J dose, significantly increases performance, decreases DOMS, and improves biochemical marker related to skeletal muscle damage.
[12]	Vanin, A.A.; De Marchi, T.; Tomazoni, S.S. et al. Pre-exercise infrared low-level laser therapy (810 nm) in skeletal muscle performance and postexercise recovery in humans. What is the optimal dose? A randomized, double-blind, placebo-controlled clinical trial. *Photomed. Laser Surg.* **2016**, *34*, 10, 473–482. doi:10.1089/pho.2015.3992.	RCT	Laser	Before	Twenty-eight high-level soccer athletes volunteer	Cluster with 5 diodes, 810 nm, 200 mW	10 J, 30 J, or 50 J on six sites, in total: 60 J 180 J 300 J	Eccentric exercise protocol	Six sites on quadriceps	Before exercise protocols, after 1 min, and 1, 24, 48, 72, and 96 h after the end of eccentric exercise protocol used to induce fatigue	MVC, DOMS, CK, and interleukin-6 (IL-6)	MVC ↑ after exercise to 24 h with 50 J MVC ↑ from 24 h to 96 h with 10 J dose PBM had no effect in decreasing DOMS. CK↓ and IL-6 ↓ with 10 J and 50 J (better results). No differences (*p* > 0.05) for 30 J dose in any of the measured results. Conclusion: Pre-exercise PBM, mainly with 50 J, significantly increases performance and improves biochemical markers related to skeletal muscle damage and inflammation.
[13]	Pinto, H.D.; Vanin, A.A.; Miranda E.F.; et al. Photobiomodulation therapy improves performance and accelerates recovery of high-level rugby players in field test: A randomized, crossover, double-blind, placebo-controlled clinical study. *J. Strength Cond. Res.* **2016**, *30*, 12, 3329–3338. doi:10.1519/JSC.0000000000001439.	COD	Laser diodes + LEDs	Before pre-exercise PBMT: 20 min before Bangsbo sprint test (BST).	Twelve males high-level rugby athletes	Cluster with 12 diodes (4 laser diodes of 905 nm, 4 light emitting diodes (LEDs) of 875 nm, and 4 LEDs of 640 nm IR + RED: 905 nm +875 nm + 640 nm	30 J per site (30 J × 17 = 510 J for each lower limb)	Three test phases, administered 1 week apart, were performed on the same day of the week (Tuesday) and time (1–5 PM)	Seventeen sites of each lower limb, i.e., quadriceps (9 points) Hamstrings (6 points) Triceps surae (2 points)	Bangsbo sprint test (BST) at familiarization phase (week 1); at weeks 2 and 3.	Perceived fatigue score (from a questionnaire); mean sprint time (ST-mean), best sprint time (ST-best) and fatigue index from Bangsbo sprint test (BST). Blood lactate levels were assessed at baseline, and at 3, 10, 30, and 60 min after BST.	Time of sprints (ST-mean) ↓ for PBMT Fatigue index during BST ↓ for PBMT Blood lactate levels ↓ in PBMT group Perceived fatigue was significantly lower in PBMT group comparatively with placebo (*p* ≤ 0.05). Conclusions: Pre-exercise PBMT with the combination of super-pulsed laser (low-level laser), red LEDs, and infrared LEDs can enhance performance and accelerate recovery of high-level rugby players in field test. This opens a new avenue for wide use of PBMT in real clinical practice in sports settings. Potential use of PBMT as a prophylactic strategy for performance and recovery enhancement of high-level athletes.
[14]	De Oliveira, A.R.; Vanin, A.A.; Tomazoni, S.S.; et al. Pre-exercise infrared photobiomodulation therapy (810 nm) in skeletal muscle performance and postexercise recovery in humans: What is the optimal power output? *Photomed. Laser Surg.* **2017**, *35*, 11, 595–603. doi:10.1089/pho.2017.4343.	RCT	Five IR laser diodes	Before the eccentric contraction protocol	Twenty-eight high-level soccer players	Cluster with 5 diodes, (810 nm) and output power of 100, 200, 400 mW per diode.	10 J/site Total = 300 J	PBMT or placebo before isokinetic exercise	Six sites of knee extensors (quadri-ceps)	Eccentric contractions of knee extensors	MVIC, DOMS, CK and LDH, inflammation (IL-1β, IL-6, and TNF-α), and oxidative stress (catalase, superoxide dismutase, carbonylated proteins, and thiobarbituric acid) were evaluated before isokinetic exercise, as well as at 1 min and at 1, 24, 48, 72, and 96 h, after the eccentric contraction protocol.	PBMT positive results for 100 mW (best) and 200 mW output power (*p* < 0.05): MVIC ↑ DOMS ↓ CK ↓ and LDH ↓ inflammation ↓ (IL-1β, IL-6, and TNF-α), and oxidative stress ↓. Conclusions: PBMT with 100 mW power output per diode (500 mW total) before exercise achieves best outcomes in enhancing muscular performance and post-exercise recovery. Higher output power is not preferable.
[15]	Rossato, M.; Dellagrana, R.A.; Sakugawa, R.L.; et al. Time response of photobiomodulation therapy on muscular fatigue in humans. *J. Strength Cond. Res.* **2018**, *32*, 11, 3285–3293. doi:10.1519/JSC.0000000000002339.	RCT	Laser/ LEDs	PBMT applied immediately before the test	Sixteen male volunteers (26 ± 6.0 years, 81 ± 12 kg, and 181 ± 7.4 cm)	Laser (850 nm), 5 diodes, 100 mW output power/each, spot size = 0.06 cm^2^, power density = 1666.6 mW/cm^2^; CW. Energy = 3.2 J/each. LED (670 nm), 12 diodes, 10 mW output power/each, spot size = 1.92 cm^2^; power density = 5.20 mW/cm^2^; CW. Energy = 0.3 J/each. LED (880 nm), 8 diodes, 25 mW output power/each, spot size = 1.28 cm^2^; power density = 19.53 mW/cm^2^; CW. Energy= 0.5 J/each. LED (950 nm), 8 diodes, 15 mW output power/each, 1.28 cm^2^; 11.71 mW/cm^2^; CW. Energy = 0.5 J/each.	30 J/site Total = 270 J	PBMT applied both 6 h before and immediately before the test.	Nine sites	Protocol in 5 sessions. Fatigue of knee extensors	Maximal isometric voluntary contraction (MIVC) was assessed before and after an isokinetic fatigue (45 flexion-extension concentric at 180°·s), associated with electromyography (root mean square [RMS] and median of frequency [MF]).	Peak torque during MIVC (pre to post) was reduced in 6 h before + immediately before treatment (26%) compared with control (33%), placebo (29%), and immediately before (32%). Conclusion: PBMT applied with 6 h + directly before exercises is capable to diminish the tiredness.
[16]	De Marchi, E.C.P.; Leal-Junior, K.C. Lando et al., “Photobiomodulation therapy before futsal matches improves the staying time of athletes in the court and accelerates post-exercise recovery,” *Lasers Med. Sci.* **2019**, *34*, 139–148. doi:10.1007/s10103-018-2643-1.	Randomized, triple-blinded, placebo-controlled, crossover clinical trial.	A cluster with 12 diodes (4 laser diodes of 905 nm, 4 LEDs of 875 nm, and 4 LEDs of 640 nm)	PBM was administered at each match before matches (40 min)	Six healthy men, futsal athletes, with an average age of 26.16 ± 6.91 years, participated in this study. The athlete’s performance was quantified according to the time on the field. The videos were analyzed to quantify the athletes spent on the field and the distance they covered.	Four super-pulsed IR Lasers 905 (±1) nm Frequency = 250 Hz. Peak power 12.5 W/each. Average mean optical output = 0.3125 mW/each. Power density = 0.71 mW/cm^2^/each. Energy density = 0.162 J/cm^2^/each. Dose = 0.07125 J/each. Spot size of laser = 0.44 cm^2^/each. Four red LEDs (nm) 640 (±10) Frequency = 2 Hz. Average optical output = 15 mW/each. Power density = 16.66 mW/cm^2^/each Energy density = 3.8 J/cm^2^/each. Dose = 3.42 J/each. Spot size of red LED = 0.9 cm^2^/each. Four IR LEDs 875(±10) nm 16 Hz Average output power 17.5 mW/each. Power density = 19.44 mW/cm^2^/each. Energy density = 4.43 J/cm^2^/each. Dose = 3.99 J/each. Spot size of LED = 0.9 cm^2^/each. Irradiation time per site = 228 s. Total dose per site = 30 J. Total dose applied per lower limb = 510 J Aperture of device = 20 cm^2^.	30 J per site	The study was conducted in two different team matches in a time interval of approximately 2 weeks between the first and second interventions.	Phototherapy was performed at nine different knee extensor and hip flexor muscle locations, six knee flexor muscle and hip extensor muscle locations, and two plantar flexor muscle locations of both lower limbs	Seventeen sites of each lower limb were irradiated. Each site of irradiation received a 30-J dose delivered in 228 s (3 min and 48 s per site).	Levels of CK, TBARS, CP, LDH, and lactate, were considered in blood samples collected before, immediately after, and 48 h after a match. The time on pitch and the distance covered by the athletes on the pitch were also taken into account. Time on pitch Moderate Distance covered	PBMT significantly increased the time of staying in the pitch and triggered a significant improvement in all the biochemical markers evaluated. No statistically significant difference was found for the mileage. Pre-exercise PBMT can successfully increase the workout and speed up the rehabilitation process.
[17]	Dornelles, M.P.; Fritsch, C.G.; Sonda, F.C.; et al. Photobiomodulation therapy as a tool to prevent hamstring strain injuries by reducing soccer-induced fatigue on hamstring muscles. *Lasers Med. Sci.* **2019**, *34*, 6, 1177–1184. doi:10.1007/s10103-018-02709-w.	COD	One hundred and fifty-two IR LEDs (880 nm)	PBMT was applied on the hamstring muscles before the simulated football match.	Twelve male amateur soccer players (~ 25 years) participated in this randomized, crossover, double-blinded, placebo-controlled trial. The volunteers were evaluated in two sessions, with a minimum 7-day interval. At each session, volunteers received either PBMT (300 J per thigh) or placebo treatment on the hamstrings prior to the simulated soccer match.	The device contains 152 infrared LEDs (880 nm) distributed evenly over an area of 252 cm^2^ (10.5 cm × 24 cm) CW Power density (mW) − each= 33 Spot size (cm^2^) each = 0.1357; Power density (mW/cm^2^) − each = 243.8	At each session, volunteers received either PBMT (300 J per thigh) or placebo treatment on the hamstrings prior to the simulated soccer match.	Volunteers performed isokinetic concentric and eccentric tests by submaximal contractions. Two sets of three maximal knee extensors concentric contractions and two sets of three maximal knee flexors were eccentric contractions conducted	The hamstrings’ muscle; quadriceps	The PBMT device used in the current study required 60 s to deliver that energy amount to each hamstring muscle (i.e., a 2 min treatment per player), so it is a feasible therapy for application before a soccer match or even during the half-time interval.	Isokinetic dynamometry and countermovement jump (CMJ) tests, respectively, before and immediately after the simulated soccer match. Quadriceps CON CMJ height (cm) Hamstring ECC H:Q functional ratio	PBMT applied immediately on the muscles of the posterior thigh before a simulated football match proves effective in attenuation of fatigue-related disorders in hamsters’ maximal eccentric resistance, H: Q resistance ratio and CMJ performance. These results advocate PBMT as an encouraging instrument to attenuate hamstring muscle tiredness and thus stopping hamstring stretch lesions that usually occur in football players.
[18]	Jówko, E.; Płaszewski, M.; Cieśliński, M.; et al. The effect of low-level laser irradiation on oxidative stress, muscle damage and function following neuromuscular electrical stimulation. A double blind, randomised, crossover trial. *BMC Sports Sci. Med. Rehabil.* **2019**, *11*, 38. doi:10.1186/s13102-019-0147-3.	COD	Laser	PBMT before NMES session.	Twenty-four moderately active, healthy men aged 21–22 years, divided into two groups.	Cluster, 4 semiconducting lasers (830 nm, 200 mW output power each)	30 J each area Total energy delivered per muscle = 180 J	PBMT or placebo before NMES, intercrossed between the 2 groups after 8-day washout period.	Six sites on quadriceps femoris muscle	Forty-five electrically evoked tetanic, isometric contractions of the quadriceps femoris (NEMS), preceded by PBMT or sham-PBMT.	MIVC—maximal (isometric) voluntary contraction; S1—pain severity measurement—pressure test; S2—pain severity measurement –squat test. Collected blood samples for: SOD in erythrocytes, the activity of GPx in the whole blood, the TAC of plasma, and the level of MDA in plasma, taken prior to the NMES session (at baseline), immediately (0), 24, 48, 72, and 96 h after NMES.	NMES-evoked contractions induced oxidative stress, increased lipid peroxidation and impairments in enzymatic antioxidant system. PBMT had a protective effect on NMES-induced decrease in enzymatic antioxidant defense and shortened the duration of inflammation. Conclusions: PBMT may protect from impairments in enzymatic antioxidant system and may shorten inflammation induced by a single NMES session in moderately active, healthy men. However, the effects of PBMT on redox state and inflammatory processes do not seem to affect muscle damage and recovery of muscle function after NMES.
[19]	Tomazoni, S.S.; Machado, C.D.S.M.; De Marchi, T.; et al. Infrared low-level laser therapy (photobiomodulation therapy) before intense progressive running test of high-level soccer players: Effects on functional, muscle damage, inflammatory, and oxidative stress markers—A randomized controlled trial. *Oxid. Med. Cell. Longev.* **2019**, 6239058. doi:10.1155/2019/6239058.	RCTtriple-blind	810 nm IR; Five-diode laser cluster CW	PBMT or placebo, the volunteers performed a standardized high-intensity progressive running test (ergospirometry test) until exhaustion. PBMT applied before a progressive running test	Twenty-two high-level male soccer players from the same team were recruited and treated with active PBMT and placebo. 5 diodes and 17 different sites were irradiated, a total of 85 points were irradiated in each lower limb, with a total of 850 J of energy delivered per lower limb (50 J per site). The study was performed in two stages, since it was a crossover study, with a washout period of 14 days between stages	810 nm IR; 5 diods. Optical output (per diode) 100 mW or 0 mW (placebo) Spot size (per diode) 0.0364 cm^2^ Power density (per diode) 2.75 W/cm^2^ or 0.00 W/cm^2^ (placebo) Energy (per diode) 10 J Energy density (per diode) 275 J/cm^2^ or 0 J/cm^2^ (placebo)	850 J (450 J on knee extensor muscles, 300 J on knee flexor muscles, and 100 J on plantar flexor muscles) Cluster area 9.6 cm^2^	High-level footballers from the same team were recruited; volunteers performed a standardized high-intensity progressive operation test (ergospirometric test)	PBMT contact with the skin at nine different sites of the knee extensor muscles (three medial, three lateral, and three central sites), six different sites of the knee flexor muscles (three medial and three lateral sites), and two different sites of the ankle plantar flexor muscles (one medial and one lateral site) of both lower limbs	Exposure time 100 s Number of irradiated sites per lower limb 9 sites on knee extensor muscles (3 medial, 3 lateral, and 3 central) 6 sites on knee flexor muscles (3 medial and 3 lateral) 2 sites on plantar flexor muscles (1 medial and 1 lateral)	Before extent and intervention (active PBMT or placebo) and then, exactly 5 min after the intense progressive run test (ergospirometry test), was analyzed rates of oxygen uptake (VO2 max) and blood samples for: CK, LDH, levels IL-1-β, IL-6, TNF-α, CAT, TBARS, SOD, carbonylated proteins	Results demonstrated that pre-exercise PBMT as a stand-alone therapy was able to improve the athletic presentation and the biochemical markers related to muscle impairment and inflammation in high-level athletes. Pre-exercise PBMT had a remarkably antioxidant effect, attenuating the oxidative stress generated by the physical activity, suggesting that this could be one of the possible mechanisms of action through which PBMT promotes ergogenic and protective effects for the skeletal muscles.
[20]	Da Cunha, R.A.; Pinfildi, C.E.; de Castro Pochini, A.; et al. Photobiomodulation therapy and NMES improve muscle strength and jumping performance in young volleyball athletes: A randomized controlled trial study in Brazil. *Lasers Med. Sci.* **2020**, *35*, 3, 621–631. https://doi.org/10.1007/s10103-019-02858-6.	RCT	850 nm 50 mW CW. NMES Burst 2 ms Frequency modulation 70 Hz.	Before undergoing strength and plyometric training.	Thirty-six athletes were included in the jump training program. All three groups (control, photobiomodulation therapy, and NMES) participated in all 18 sessions, for a total of 648 sessions. In addition, no athletes were lost in the follow-ups at 6 or 8 weeks.	Total average radiant power 150 mW. Radiant energy per diode 2 J. Radiant energy per point 6 J. NMES Duty cycle 10% T-on 10 s T-off 30 s. Intensity maximum tolerable	Total radiant energy 36 J.	Jump training	PBM was applied bilaterally and continuously immediately before the six-seat training session on the surface of the femoral quadriceps muscles. The quadriceps femoris muscle was electrically elicited with two self-adhesive electrodes (8 × 13 cm, for an area of 104 cm^2^). One electrode was positioned on the rectus femoris muscle, 20 cm from the anterior superior iliac spine; the other was positioned on the oblique vastus medialis muscle, 5 cm from the patella superior pole.	Exposure duration laser per point 40 s. Output power 290 mW. Energy density per point 0.8 J/cm^2^. NMES T-on 10 s. The NMES group additionally underwent NMES-based quadriceps femoris muscle strength training (base frequency 1 kHz, frequency modulation 70 Hz, intensity maximum tolerable).	The following parameters were analyzed: dominant strength of the lower limbs (N/kg), jumping capacity (cm), jumping frequency (n) and general impression (−5 to +5). Baseline (pre-training), post-workout (6 weeks) and 8-week follow-up for the control group, for PBMT and NMES.	This study found that, for volleyball athletes, PBMT and NMES both promoted benefits in terms of muscle-strength gain. These benefits were maintained for 2 weeks even after training was interrupted. Dominant lower limb strength improved in the NMES group compared to the control. Non-dominant lower limb strength increased in both the PBMT group and the NMES group compared to the control group, but the NMES group improved significantly more than the PBMT group; the NMES group also improved in global impression of jumps compared to the control group.
[21]	Rossato, M.; Dellagrana, R.A.; Sakugawa, R.L.; et al. Dose-Response Effect of Photobiomodulation Therapy on Muscle Performance and Fatigue During a Multiple-Set Knee Extension Exercise: A Randomized, Crossover, Double-Blind Placebo-Controlled Trial. *Photobiomodul. Photomed. Laser Surg.* **2020**, *38*, 12, 758–765. doi:10.1089/photob.2020.4820.	Crossover double-blind RCT.	Cluster with 5 lasers and 28 LEDs, as follows: 5 IR lasers (850 nm), Twelve red LEDs (670 nm), 8 IR LEDs (880 nm) and 8 IR LEDs (950 nm).	PBM 6 h before and immediately before the exercise protocol.	Eighteen (26 ± 6.0 years; 82 ± 11 kg; and 186 ± 7.3 cm) physically active men were evaluated and a minimum of 15 subjects were randomised to investigate the effects of PBMT versus the placebo.	Five lasers (850 nm, 1666.6 mW/cm^2^), 12 LEDs (670 nm, 5.20 mW/cm^2^), 8 LEDs (880 nm, 19.53 mW/cm^2^), and 8 LEDs (950 nm, 11.71 mW/cm^2^), CW. Exposure duration: 16, 32, or 64 s; Radiant exposure = 0.9933 J/cm^2^; Radiant energy: 15, 30, or 60 J. Number of points irradiated = 9 Area irradiated = 30.2 cm^2^. Application technique: cluster.	Total energy over the entire treatment course 270, 540, or 1080 J.	Isokinetic exercise protocol (5 sets of 10 knee extension repetitions, maximum contractions at 60° s^−1^) in 6 sessions, one week apart. Control condition (no PBMT/placebo treatments) was applied at the first and sixth sessions.	Nine sites on quadriceps.	Placebo or PBMT with 135, 270, or 540 J/quadriceps was randomly applied from the second to fifth sessions.	The isometric and isokinetic concentric peak torques were assessed before and after the exercise protocol.	Knee extension exercise performance was not affected by PBMT compared with placebo. All PBMT doses led to likely positive effects on isometric peak torque (IPT), concentric peak torque (CPT), and concentric work (W) compared to placebo. Conclusion: PBMT with 135, 270, and 540 J applied 6 h before and immediately before exercise was efficient to produce the same total work with lower fatigue, facilitating possible additional sets for performance (i.e., higher workout volume).
				**After**								
[22]	Zagatto, A.M.; de Paula Ramos, S.; Nakamura, F.Y.; et al. Effects of low-level laser therapy on performance, inflammatory markers, and muscle damage in young water polo athletes: A double-blind, randomized, placebo-controlled study. *Lasers Med. Sci.* **2016**, *31*, 3, 511–521. doi:10.1007/s10103-016-1875-1.	RCT	Laser	PBM 5–40 min immediately after each daily training session.	Twenty young male water polo players (15.4 ± 1.2 years, body mass 68.3 ± 10.5 kg, height 173.9 ± 5.9 cm, and body mass index 22.5 ± 2.6 kg/m^2^).	810 nm.	48 J (i.e., 24 J per leg).	Training routine: 6 days per week, 4 h sessions per day: 2 h of swimming training and 2 h of water polo training, except Saturday—match simulation.	Eight points on the adductor magnus and adductor longus muscles (spot size area of 0.028 cm^2^) = 16 points.	Pre- and post- PBM, for 5 days.	Performance: was measured by a 200-m maximal swimming (P200) and a 30-s crossbar jump test (30 CJ) every day before training, and blood samples pre- and post- PBM, to measure interleukins (IL) (i.e., interleukin-1 beta, interleukin-10, and tumor necrosis factor-alpha) and muscle damage markers (i.e., CK and LDH).	No significant change in the P200 exercise Moderate improvement in the 30 CJ (8.7 ± 2.6 %). IL-1β↑ and TNF-alpha↑ (*p* < 0.016) 48 h after last PBMT, compared to pre-, 0, and 24 h, but did not differ between groups. IL-10↑ increased over time in the placebo group and reached a moderate effect compared to the PBM group. CK↓ significantly (*p* = 0.049) over the time in the PBM group, but there was no significant change in LDH (*p* = 0.150). Conclusion: PBM resulted in small to moderate effect on inflammatory and muscle damage markers, and a moderate effect on performance in water polo players. Lack of positive results could be due to the small area covered by irradiation, and this should be considered in future studies.
[23]	De Paiva, P.R.; Tomazoni, S.S.; Johnson, D.S.; et al. Photobiomodulation therapy (PBMT) and/or cryotherapy in skeletal muscle restitution, what is better? A randomized, double-blinded, placebo-controlled clinical trial. *Lasers Med. Sci.* **2016**, *31*, 9, 1925–1933. doi:10.1007/s10103-016-2071-z.	RCT	One laser diode + 8 LEDs	Three minutes after high-intensity eccentric contractions.	Fifty healthy male volunteers were randomized into 5 groups (PBMT, cryotherapy, cryotherapy + PBMT, PMBT + cryotherapy, or placebo).	Cluster with one IR laser diode (905 nm), 4 red LEDs (640 nm) and 4 IR LEDs (875 nm).	Total energy per limb: 240 J.	Treatments repeated at 24 h, 48 h and 72 h.	Six points per limb, as follows: Two points on vastus medialis Two points on vastus laterallis Two points on rectus femoris.	Eccentric contractions of knee extensor muscles in an isokinetic dynamometer.	Post-exercise assessments: Exercise performance (maximum voluntary isometric contraction MVIC). Delayed onset muscle soreness (DOMS). Muscle damage (CK). Pain on visual analogue scale (VAS). Assessments were performed at baseline; immediately after; and at 1 h, 24 h, 48 h, 72 h, and 96 h.	PBMT alone was optimal for post-exercise recovery with: MVIC ↑, DOMS ↓, CK activity ↓ (*p* < 0.05) from 24 h to 96 h compared to placebo, cryotherapy, and cryotherapy + PBMT. In the PBMT + cryotherapy group, the effect of PBMT was decreased (*p* > 0.05) but demonstrated significant improvement in MVIC↑, DOMS↓, CK activity↓ (*p* < 0.05). Cryotherapy as single treatment and cryotherapy + PBMT were similar to placebo (*p* > 0.05). Conclusion: PBMT used as single treatment is the best modality for enhancement of post-exercise restitution, leading to complete recovery to baseline levels from 24 h after high-intensity eccentric contractions.
[24]	De Marchi, T.; Schmitt, V.M.; Machado, G.P.; et al. Does photobiomodulation therapy is better than cryotherapy in muscle recovery after a high-intensity exercise? A randomized, double-blind, placebo-controlled clinical trial. *Lasers Med. Sci.* **2017**, *32*, 2, 429–437. doi:10.1007/s10103-016-2139-9.	RCT	LEDs	PBMT and/or cryotherapy was applied, 2 min after post-exercise MVC test.	Forty volunteers were randomly divided into five groups: placebo group (PG); PBMT group (PBMT); cryotherapy group (CG); cryotherapy-PBMT group (CPG); and PBMT-cryotherapy group (PCG).	Cluster of 69 LEDs (34 red LEDs and 35 infrared LEDs), with 660 nm and 850 nm, 10 mW (red) and 30 mW IR output power (each diode).	Total = 41.7 J (for 30 s of irradiation)	PBMT or placebo after muscle fatigue-inducing protocol (MFIP). Application of ice was limited to 20 min.	Cluster (69 sites) on the muscle belly of the biceps, i.e., elbow flexors (biceps brachii).	Four sessions of MFIP at 24 h intervals. Exactly 30 s after the MFIP, volunteers were subjected to a new MVC following the parameters of the MVCs prior to MFIP. The value found in this isolated MVC will be considered the maximum capacity of power generation of the volunteer after the exercise (post-MVC). MVC was evaluated 24 h (MVC24), 48 h (MVC48), and 72 h (MVC72) after the execution of MFIP.	Isometric assessment (MVC) and blood collection in the pre-exercise period, and after 5- and 60-min post-exercise, and 24 h, 48 h, and 72 h later.	MVC↑ in PBMT, CPG, and PCG in comparison with both PG and CG (*p* < 0.05). Significant decrease in the concentrations of the biochemical markers of oxidative damage in all groups and CK↓ in the PBMT, PCG, and CPG compared with the PG (*p* < 0.01). Conclusion: Use of PBMT is more effective than the use of cryotherapy for muscle recovery, additionally cryotherapy decreases PBMT efficacy.
[25]	Vassão P.G.; Baldini, G.S.; Vieira, K.V.S.G.; et al. Acute Photobiomodulation Effects Through a Cluster Device on Skeletal Muscle Fatigue of Biceps Brachii in Young and Healthy Males: A Randomized Double-Blind Session. *Photobiomodul. Photomed. Laser Surg.* **2020**, *38*, 12, 773–779. doi:10.1089/photob.2019.4786.	RCT double blind.	Fourteen LEDs, as follows: 7 red diodes (630 nm) and 7 IR diodes (850 nm).	PBM immediately after the fatigue protocol.	Thirty-two males aged 18 to 25 years, clinically healthy, and classified as active were randomized into the control group (CG), red PBM group (RPG), and infrared PBM group (IPG).	A PBM cluster device with: 7 red diodes (630 nm), 7 IR diodes (850 nm), 100 mW/diode, 2 W/cm^2^ power density; 91 J/cm^2^ energy density; 4 J per point; 28 J total energy, 40 s.	28 J total energy per application.	PBMT with red LEDs, or IR LEDs, or placebo.	The cluster device was positioned 2 cm above the elbow joint, covering the biceps brachii surface of the dominant upper limb.	Fatigue protocol consisted of a maximum voluntary isotonic contraction of elbow flexion–extension with 75% of one-repetition maximum until exhaustion.	Muscle fatigue was analyzed by surface electromyography (EMG) recorded from the long head of biceps brachii, blood lactate concentration, and evaluation of the rate of perceived exertion (RPE) using the Borg Scale. Electromyography fatigue index (EMGFI) was calculated from EMG data.	EMGFI ↓ in the CG; RPE ↑ and lactate concentration ↑ significantly in all groups. Conclusion: Electromyography fatigue index delta value was greater in the IPG compared with the CG, suggesting that infrared could be more effective than red in decreasing muscle fatigue.
[26]	De Carvalho, G.; Gobbi, A.; Gobbi, R.B.; et al. Photobiomodulation by light emitting diode applied sequentially does not alter performance in cycling athletes. *Lasers Med. Sci.* **2020**, *35,* 8, 1769–1779. doi:10.1007/s10103-020-02973-9.	RCT	LEDs	PBMT on the second, third, and fourth day of collection, 24 h after data collection at baseline.	Forty-eight Cyclists (male, mean age 33.77 years), divided into 4 groups: IR RED IR + RED Placebo	Special cluster designed with dimensions of 25 × 42 cm^2^, with equidistant distribution between the LEDs (1 × 1 cm): Infrared (IR 940 ± 10 nm), red (RED 620 ± 10 nm), mixed Red, and IR (RED/IR 620 + 940 nm).	180 J per thigh, in total = 360 J		PBMT with infrared (IR 940 ± 10 nm), red (RED 620 ± 10 nm), mixed Red, and IR (RED/IR 620 + 940 nm) on 3 consecutive days, applied to the quadriceps femoris bilaterally.	Isokinetic contraction test. Exhaustion test.	Isokinetic dynamometer test. Incremental test until volitional exhaustion; maximum oxygen consumption (VO_2_max). Concentration of blood lactate. Thermographic evaluation 10 min before the test. Reevaluations were performed 24 h after the last application, with 1 week of follow-up.	Peak torque↑ in IR/RED group compared with sham, 24 h after the last application. A large effect size was observed for total time to exhaustion (ES = 1.98) and for VO_2_max (ES = 6.96), and a moderate effect size was seen for anaerobic threshold (ES = 0.62) in the IR/RED group, compared with sham. Conclusions: PBMT, when not associated with training, was not able to produce a cumulative effect on the performance of cycling athletes. However, the association of two wavelengths seems to be better for increased performance.
				**Before and After**								
[27]	Vanin, A.A.; Miranda, E.F.; Machado, C.S.; et al. What is the best moment to apply phototherapy when associated to a strength training program? A randomized, double-blinded, placebo-controlled trial: Phototherapy in association to strength training. *Lasers Med. Sci.* **2016**, *31*, 8, 1555–1564. doi:10.1007/s10103-016-2015-7. Erratum in: *Lasers Med. Sci.* **2017**, *32*, 253. https://doi.org/10.1007/s10103-016-2121-6.	RCT	Laser + LEDs	PBM either before and/or after each training session.	Forty-eight male volunteers mean age of 26 years old (± 5.24), height of 174.5 cm (± 7.59), and body mass 76.5 kg (± 10.8) divided in 4 groups (12 volunteers per group): GA: PBM before and after GB: PBM before, placebo after GC: Placebo before and PBM after GD: Placebo before and after.	Cluster probes with four super-pulsed laser diodes of 905 nm, four LEDs of 875 nm, and four LEDs of 640 nm	30 J (0.285 J of 905 nm, 13.68 J of 640 nm, 15.96 J of 875 nm) in total treatment time of 228 s (3 min and 48 s).	Twelve weeks strength training protocol in Leg extension and Leg press (5 × 10 repetitions 80% of 1-RM), 2 sessions a week on non-consecutive days (72 h of rest) for 12 consecutive weeks (total of 24 training sessions) and the workload was adjusted by retesting the 1-RM test at 4th and 8th week.	Six different sites of the anterior muscle of the thigh (two centrally—rectus femoris and vastus intermedius, two laterally—vastus lateralis, and two medially—vastus medialis) for both legs.	Baseline, 4 weeks, 8 weeks, and 12 weeks. Time length between irradiation and the exercise protocol both to pre- and post-treatments was 5 to 10 min.	Peak torque reached in MVC test, load in 1-RM test and thigh circumference (perimetry) at larger CSA.	No significant differences (*p* > 0.05) at baseline for all 4 groups for MVC, 1-RM test, or perimetry. No significant differences (*p* > 0.05) were observed between groups for any experimental time regarding perimetry. MVC↑, both in absolute and percentages, (*p* < 0.05) by pre-exercise PBM. 1-RM test ↑, with the leg press and leg extension. Conclusions: Pre-exercise PBM increased muscle strength in the case of training twice a week for 12 weeks. PBM before exercise increased isometric strength in 39% to 46%, while only 14% to 15% in the placebo group.
[28]	Felismino, A.S.; Costa, E.C.; Aoki, M.S.; et al. Effect of low-level laser therapy (808 nm) on markers of muscle damage: A randomized double-blind placebo-controlled trial. *Lasers Med. Sci.* **2014**, *29*, 933–938. doi:10.1007/s10103-013-1430-2.	RCT	IR Laser	Between the sets of the biceps curl exercise.	Twenty-two physically active men were randomized into two groups: placebo (n = 11) and laser (n = 11).	Laser (808 nm; 100 mW; 35.7 W/cm^2^, 357.14 J/cm^2^ per point, 10 s), or placebo.	1 J per point applied for 10 s on four points of the biceps brachii of each arm.	Exercise-induced muscle damage protocol for biceps brachii (biceps curl, 10 sets of 10 repetitions with load of 50% of one-repetition maximum test (1 RM).	Arms muscles.	1. All volunteers were submitted to one-repetition maximum test (1 RM) of elbow flexion-extension (biceps curl) (visit 1); 2. Seven days after the 1 RM test, all volunteers returned to the laboratory and were measured the CK levels in the blood (plasma). Subsequently, they performed the exercise-induced muscle damage protocol plus laser irradiation (active or placebo) between the sets of this protocol on both upper limbs (visit 2); 3. Re-measurement of CK levels in the blood and the strength performance test (1 RM) after the visit at 24 h (visit 3), 48 h (visit 4), and 72 h (visit 5).	Creatine kinase (CK) activity and maximum strength performance (1 RM) were measured before, immediately after, 24 h, 48 h, and 72 h after the exercise-induced muscle damage protocol.	CK ↑ after the muscle damage protocol in both groups; the increase was diminished in the laser group compared to the placebo group at 72 h. (1 RM) ↓immediately after the muscle damage protocol in both groups, but it returned to the baseline level in both groups at 24 h, 48 h, and 72 h. Conclusion: CK activity was reduced 72 h after the muscle damage protocol in laser group, but no obvious positive effect on strength performance recovery was observed.
[29]	de Brito Vieira, W.H.; Bezerra, R.M.; Queiroz, R.A.; et al. Use of low-level laser therapy (808 nm) to muscle fatigue resistance: A randomized double-blind crossover trial. *Photomed. Laser Surg.* **2014**, *32*, 12, 678–685. doi:10.1089/pho.2014.3812.	RCT with crossover design.	IR Laser device (808 nm).	LLLT applied before or after intense exercises.	Seven young men (21 ± 3 years of age) who were clinically healthy, were allocated randomly into two groups: Active laser (LLLT) and placebo laser (Placebo).	Laser device (808 nm, CW, 100 mW). Spot size = 0.0028 cm^2^. Power density = 35.71 W/cm^2^. Treatment time per point = 40 s. Energy per point = 4 J. Energy density = 1428.57 J/cm^2^ Number of irradiation points per muscle: 3 points (rectus femoris); 1 point (vastus medialis); 1 point (vastus lateralis). Total energy delivered per muscle: 12 J (rectus femoris) × 3 times = 36 J. 4 J (vastus medialis) × 3 times= 12 J. 4 J (vast:us lateralis) × 3 times= 12 J.	4 J per point.	3 × 20 RM of knee flexion-extensions.	LLLT in contact mode and perpendicular to the belly of quadriceps femoris muscles at 5 equidistant points. After 1 week (washout period), all volunteers were exchanged among groups and then all assessments were repeated.	Three sets of 20 maximum repetitions (RM) of knee flexion-extension at 60 degrees/s using an isokinetic dynamometer. During rest intervals (between sets of exercise), LLLT or Placebo was applied perpendicularly on the quadriceps femoris muscles exactly over the same points where electrodes were placed for EMG.	Maximum repetitions (RM) using the isokinetic dynamometer was evaluated at 60 degrees/s until exhaustion or fatigue; that is, at the moment that participants were not able to keep muscle contraction throughout a preset range of motion (75 degrees of knee flexion-extension) or when the participants made a voluntary decision to stop. EMG analysis: root mean square (RMS) and median frequency (MF) as two EMG parameters commonly used to conclude muscle fatigue. Electromyography fatigue index (EFI). Heart rate (HR) was recorded at rest and during maximal effort.	LLLT ↑ the maximum number of RM, comparatively with control group. For both groups, MF ↓ significantly for all muscles, comparing pre and post evaluations at baseline and end point. HR between groups had no statistical significance. Conclusion: LLLT increased RM and reduced EFI, compared with the placebo group, helpful for high performance that demand fast return to a normal state and less tiredness.
				Laboratory Settings								
[30]	Florianovicz V.C.; Ferraresi, C.; Kuriki, H.U. et al. Effects of Photobiomodulation Therapy and Restriction of Wrist Extensor Blood Flow on Grip: Randomized Clinical Trial. *Photobiomodul. Photomed. Laser Surg.* **2020**, *38*, 12, 743–749. doi:10.1089/photob.2019.4800.	RCT, but not double-blind.	Laser equipment (red 660 nm and infrared 830 nm).	PBMT was applied before (approx. 10 min) each workout.	Fifty-eight volunteers (clinically healthy women, aged 18–25 years old) divided into 4 groups: (1) control (2) BFR (strengthening with blood flow restriction), (3) 660 nm + BFR (4) 830 nm + BFR.	660 nm PBMT— 35 mW; 0.05 cm^2^; 2.10 J/per point; Total energy = 18.9 J Power density = 700 mW/cm^2^ 830 nm PBMT— 32 mW; 0.101 cm^2^; 1.92 J/per point; Total energy = 17.2 J. Power density = 316.8 mW/cm^2^	Time of irradiation = 60 s Energy density 42 J/cm^2^ (red); 19 J/cm^2^ (IR).	Hypothesis: PBMT + BFR would increase muscle strength gain. 10 sessions: one evaluation session, 8 intervention sessions, and one reevaluation.	Average pressure to promote BFR, as follows: 1. BFR group: 140 ± 12.79 mmHg; 2. 660 nm group 133 ± 6.22 mmHg; 3. 830 nm group 128 ± 8.7 mmHg.	One repetition maximum (1 RM) = largest load that volunteer could perform with complete wrist extension, starting from total wrist flexion. Electromyography (EMG) was performed during the grip strength task.	Handgrip strength, wrist extensor muscle strength, and electromyography (EMG) of the radial carpal extensor muscle.	Wrist extensor strength significantly increased for both the 660 nm group (baseline 6.24 ± 0.84; after 7.77 ± 0.58 kgF) and the BFR group (baseline 6.02 ± 0.84; after 7.54 ± 0.92 kgF) compared with the control group. Conclusion: 660 nm PBMT + BFR was effective in increasing the handgrip strength of wrist extensor muscles, as well as increasing muscle recruitment in healthy subjects.
[31]	Miranda, E.F.; Vanin, A.A.; Tomazoni, S.S.; et al. Using pre-exercise photobiomodulation therapy combining super-pulsed lasers and light-emitting diodes to improve performance in progressive cardiopulmonary exercise tests. *J. Athl. Train* **2016**, *51*, 2, 129–35. doi:10.4085/1062-6050-51.3.10.	RCT with crossover design.	Four laser diodes + 8 LEDs	Immediately before a progressive cardiopulmonary test.	Twenty untrained male volunteers. received active PBMT, or placebo at session 1, and the other treatment at session 2.	Cluster with: 4 IR laser diodes (905 nm) 4 IR LEDs (875 nm) 4 red LEDs (640 nm)	30 J per site Total energy = 30 × 17 = 510 J	PBMT or placebo on 2 visits, 1 week apart. A progressive cardiopulmonary exercise test was performed on a motor-driven treadmill 5 to 10 min after each therapeutic administration.	Seventeen sites on each lower limb (9 on the quadriceps, 6 on the hamstrings, and 2 on the gastrocnemius muscles).	A progressive cardiopulmonary treadmill exercise test: running on treadmill until exhaustion.	Distance covered. Time to exhaustion. Ventilatory rate. Dyspnea.	PBMT effects: distance covered ↑, time to exhaustion ↑, ventilatory rate ↑. dyspnea ↓. Conclusion: PBMT with super pulsed lasers and LEDs applied before a progressive cardiopulmonary exercise test on a treadmill increased distance covered, time to exhaustion, and pulmonary ventilation, and decreased dyspnea in healthy volunteers.

**Table 2:** PBM in human studies without effects.

No	References/Year	Type of Study	Type of Light/Devices	PBM Before/After Activity	Total Energy (J) Applied	Types of Physical Activities	Stimulated Muscles	Analyzed Parameters	Conclusions
[38]	Dutra, Y.M.; Claus, G.M.; Malta, E.S.; et al. Photobiomodulation 30 min or 6 h prior to cycling does not alter resting blood flow velocity, exercise-induced physiological responses or time to exhaustion in healthy men. *Front. Physiol.* **2021**, *15*, 11, 607302. doi:10.3389/fphys.2020.607302.	RCT	Multi diode array	Before	152 J	Cycling	Quadriceps, hamstrings, gastrocnemius muscles	Plasma nitrogen CK and lactate	PBMT did not improve exercise-induced changes in cardiorespiratory responses or metabolic blood markers or time to exhaustion during the severe intensity cycle performed by untrained men.
[39]	Malta, E.S.; de Lira, F.S.; Machado, F.A.; et al. Photobiomodulation by led does not alter muscle recovery in-dicators and presents similar outcomes to cold-water immersion and active recovery. *Front. Physiol.* **2019**, *14*, 9, 1948. doi:10.3389/fphys.2018.01948.	Double-blind, randomized, and placebo-controlled design	Cluster multi-diode containing 104 LED 56 diodes 660 nm and 48 diodes 850 nm.	Before	600 J (300 J per foot in 5 spots)	High-intensity interval training Cold water immersion	Two regions of the quadriceps muscle, two regions of the biceps femoris, and one region between the soleus and gastrocnemius muscles.	IL-10, TNFα, CK; LDH, DOMS, CMJ, SIT	PBMT had no effect on inflammation, muscle injury, CMJ, DOMS or performance after two consecutive sprint interval training sessions compared to placebo, CWI and AR strategies.
[40]	Orssatto, L.B.R.; Detanico, D.; Kons, R.L.; et al. Photobiomodulation therapy does not attenuate fatigue and muscle damage in judo athletes: A randomized, triple-blind, placebo-controlled trial. *Front. Physiol.* **2019**, *10*, 811. doi:10.3389/fphys.2019.00811.	Randomized, Triple-Blind, Placebo-Controlled Trial.	LASERs (850 nm) LEDs (670 nm) LEDs (880 nm) LEDs (950 nm) Number of Diodes = 33 (5, 12, 8, 8)	Before	Dose per site = 30 J. Total dose = 450 J Quadriceps = 240 J, Hamstrings = 120 J, Gastrocnemius = 60 J.	Sixteen judo athletes	Fifteen sites on each lower limb: eight sites on the quadriceps (three sites on vastus lateralis, three sites on rectus femoris, and two sites on vastus medialis), four sites on the hamstrings (two on semitendinosus and two on semimembranosus), two sites on the gastrocnemius (one on lateralis and one on mediallis areas), and one site on the soleus.	CMJ (impulse, peak power, peak velocity, and peak force). Rate of perceived exertion, fatigue, and muscle soreness.	No effect of PBM used before exercise to reduce lower limb muscle fatigue and damage during and following a stretch-shortening cycle protocol in judo athletes.
[41]	Orssatto, L.B.R.; Rossato, M.; Vargas, M.; Diefenthaeler, F.; de la Rocha Freitas, C. Photobiomodulation therapy effects on resistance training volume and discomfort in well-trained adults: A randomized, double-blind, placebo-controlled trial. *Photobiomodul. Photomed. Laser Surg.* **2020**, *38*, 12, 720–726. doi:10.1089/photob.2019.4777.	Randomized crossover design, placebo-controlled and double-blind. 14 well-trained adults. Visited laboratory three times, 7 days apart.	Cluster: 5 Lasers and 28 LEDs. Pulse mode (CW) 5 Lasers (850 nm), Irradiance at target: 1666.6 mW/cm^2^; 12 LEDs (670 nm), 5.20 mW/cm^2^; 8 LEDs (880 nm), 19.53 mW/cm^2^; 8 LEDs (950 nm), 11.71 mW/cm^2^; Exposure duration: 64 s; Radiant exposure: 0.9933 J/cm^2^.	Before	60 J per site, 6 sites per limb, on each calf. Total dose = 360 J per calf. Area irradiated: 30.2 cm^2^. Second and third visits, subjects were randomly submitted to PBMT.	In the first visit, 12-repetition maximum (12-RM) test was performed unilaterally on the standing calf raise machine. Resistance training session, performed unilaterally with six sets of repetitions to concentric failure.	The gastrocnemius and soleus muscle.	Rate of perceived exertion for discomfort (RPE-D). Force reduction. Repetitions fatigue index. Total repetitions volume.	PBMT has not been helpful in increasing volume or reducing discomfort during resistance training and conducted to concentric failure to well-trained participants.
[42]	Dutra, Y.M.; Claus, G.M.; Malta, E.S.; et al. Acute photobiomodulation by LED does not alter muscle fatigue and cycling performance. *Med. Sci. Sports Exerc.* **2020**, *52*, 11, 2448–2458. doi:10.1249/MSS.0000000000002394. Erratum in: *Med. Sci. Sports Exerc.* **2021**, *53*, 5, 1099. https://doi.org/10.1249/MSS.0000000000002644.	Pseudorandomized and balanced, crossover design.	18 × 38 cm matrix with 200 diodes	Before	260 J and 130 J	Cycling	-	Blood lactate concentrations, respiratory responses, EMG activity and capillary gasometry.	PBMT at doses of 260 J and 130 J had no beneficial effects on muscle fatigue, cyclic performance, metabolic parameters and muscle activity in men during recreational cycling.
[43]	Dos Santos, I.A.; Lemos, M.P.; Coelho, W.V.H.M.; et al. Acute photobiomodulation does not influence specific high-intensity and intermittent performance in female futsal players. *Int. J. Environ. Res. Public Health* **2020**, *17*, 19, 7253. doi:10.3390/ijerph17197253.	Randomized cross-over, placebo-controlled and double-blind design,	Diodes: 69: 34 diodes 660 nm and 35 diodes 850 nm CW	Before	Energy density = 4.5 J/cm^2^ (energy dose: 200 J).	Amateur female futsal players	Fifteen minutes of PBMT (1 min 30 s each muscular point; five muscular points in each lower limbs).	CMJ, blood lactate concentration, SmO2, HR, RPE for the YYIR1 test.	PBMT used before high-intensity and intermittent exercise did not influence performance, physiological and perceptual responses in amateur female futsal players.
[50]	Medeiros, D.M.; Aimi, M.; Vaz, M.A.; et al. Effects of low-level laser therapy on hamstring strain injury re-habilitation: A randomized controlled trial. *Phys. Ther. Sport* **2020**, *42*, 124–130. doi:10.1016/j.ptsp.2020.01.006.	Randomized controlled trial	Cluster probe consisting of five infrared diodes (850 nm, CW, 100 mW, density energy per diode = 206.9 J/cm^2^	After	90 J/leg	Male athletes	Three sites; biceps femoris, medial hamstring 60 s.; 90 J/leg	PSLR, KET, SLR, MHFAKE Injury grade I and II; injury site (proximal, distal) strength deficit, Pain.	PBM did not improve functional rehabilitation after HSI in amateur athletes after an exercise program.
[56]	Ghigiarelli, J.J.; Fulop, A.M.; Burke, A.A.; et al. Effects of whole-body photobiomodulation light-bed therapy on creatine kinase and salivary interleukin-6 in a sample of trained males: A randomized, crossover study. *Front. Sports Act Living* **2020**, *29*, 2, 48. doi:10.3389/fspor.2020.00048.	Randomized, counterbalanced, cross-over design	Mixed, 660 and 850 nm, with 2800 diodes	Before and after	Total energy emitted over 15 min period = 473 J and 400 J.	Trained males underwent an exercise-induced muscle-damaging training session.	Bench Press; chin-ups Repeated sprints Cycling	CK; salivary IL6	PBMT did not significantly reduce the activity of salivary IL-6 or CK concentration during post-intensity recovery endurance training for 24 to 72 h.
[66]	Malta, E.D.S.; De Poli, R.A.; Brisola, G.M.; et al. Acute LED irradiation does not change the anaerobic capacity and time to exhaustion during a high-intensity running effort: A double-blind, crossover, and place-bo-controlled study: Effects of LED irradiation on anaerobic capacity and performance in running. *Lasers Med. Sci.* **2016**, *31*, 7, 1473–1480. doi:10.1007/s10103-016-2011-y.		104 diodes (56 diodes of 660 nm and 48 diodes of 850 nm) Frequency 0–1500 Hz Optical output 10 mW (660 nm) and 30 mW (850 nm)	After a high-intensity running effort	600 J (300 J per leg) 1.5 J/cm^2^ from each red LED and 4.5 J/cm^2^ from each infrared LED 30 s at each point	Fifteen moderately active and healthy males underwent a graded exercise test and two supramaximal exhaustive efforts at 115% of the intensity associated with maximal oxygen uptake performed after acute LEDT or placebo	Two regions of the quadriceps muscle, two regions of the biceps femoris, and one region between the soleus and gastrocnemius muscles following the distribution axis of the muscle fibers in both legs.	RPE, MAOD_ALT_ VO_2MAX_ iVO_2MAX_ REREX; HREX La − P RPEEX	PBMT after a high-intensity running effort did not alter the MAOD_ALT_, metabolic energy pathways, or high-intensity running performance.
[67]	Peserico, C.S.; Zagatto, A.M.; Machado, F.A. Effects of endurance running training associated with photo-biomodulation on 5-km performance and muscle soreness: A randomized placebo-controlled trial. *Front. Physiol.* **2019**, *10*, 211. doi:10.3389/fphys.2019.00211.	Randomized, placebo-controlled study	LEDs: 56 red diodes (660 nm) and 48 IR (850 nm).	Before all endurance training sessions.	Five points per leg, 60 J at each point, and a total energy of 300 J/per leg.	Thirty untrained subjects in 5-km performance test.	PBMT on 2 sites of the quadriceps muscle, also 2 on the biceps femoris, and one on the gastrocnemius muscle, for 30 s each point.	V-peak test, t-lim test, and 5-km running performance for both groups at pre- and post-training.	Inferential analysis did not show clear significant differences in Vpeak and t5-km for PBM group compared to placebo, and only a moderate effect in relieving muscle pain in the third week of training.
[68]	Zagatto, A.M.; Dutra, Y.M.; Lira, F.S.; et al. Full Body Photobiomodulation Therapy to Induce Faster Muscle Recovery in Water Polo Athletes: Preliminary Results. *Photobiomodul. Photomed. Laser Surg.*, **2020**, *38*, 766–772. https://doi.org/10.1089/photob.2020.4803.	Randomized, parallel, and double-blinded design.	Full body PBMT 6 panels of 76 red (660 nm, 80 mW each at 30 cm of distance) and 74 IR (850 nm, 65 mW each at 30 cm of distance) LEDs, totaling 900 LEDs distributed over an area of 12,193 cm^2^ = 1.2193 m^2^	Immediately after official water polo matches	Total average radiant power (mW) = (80 + 65) = 145. Total average power density (mW/cm^2^) (25.47 + 20.70) = 46.17 Total average energy density (J/cm^2^) (3.8 + 3.1) = 6.9	Thirteen water polo athletes (whole team).	Full body PBMT, 30 cm far from the device; irradiation time in continuous mode = 5 min (2 min 30 s to front plus 2 min 30 s back).	Before each match (2–3 h): heart rate variability (HRV) in rest; blood samples for testosterone and cortisol; CK, LDH; TNF-a, IL-6; MVC and squat jump.	Full body PBMT did not induce faster recovery of inflammatory, muscle damage (excepting LDH), testosterone, cortisol, HRV, and neuromuscular responses during repeated days of water polo matches.
[69]	Segabinazi Peserico, C.; Garozi, L.; Zagatto, A.M.; et al. Does Previous Application of Photobiomodulation Using Light-Emitting Diodes at Different Energy Doses Modify the Peak Running Velocity and Physiological Parameters? A Randomized, Crossover, Double-Blind, and Placebo-Controlled Study. *Photobiomodul. Photomed. Laser Surg.* **2020**, *38*, 12, 727–733. doi:10.1089/photob.2019.4791.	Randomized, crossover, double-blind, placebo-controlled. 15 physically active males divided into 4 groups: placebo (PLA) and 3 PBM groups with different application doses: PBM1: 30 J per area PBM2: 120 J per area PBM3: 180 J per area	Fifty-six red diodes (660 nm); 50 mW/cm^2^ 1.5 J/cm^2^ each diode; and 48 IR diodes (850 nm); 150 mW/cm^2^ 4.5 J/cm^2^ each diode.	Five minutes before treadmill tests.	Cumulative dose (on body) 300 J, 1200 J, or 1800 J; Application time (per point): 15, 60, or 90 s. Number of irradiation points (per leg) 5 points. Number of irradiation points (on body) 10 points. Total application time 75, 300, or 450 s.	Treadmill tests for the determination of Vpeak.	Two regions of the quadriceps muscle, two regions of the femoral biceps muscle, and one region of the gastrocnemius muscle in both legs.	HRmax, maximal heart rate; LApeak, peak blood lactate concentration; RPEmax, maximal rating of perceived exertion; Vpeak, peak running velocity.	Application of different doses of PBM using LEDs did not modify Vpeak and physiological and perceptual parameters.
[70]	Dellagrana, R.A.; Rossato, M.; Orssatto, L.B.R. et al. Effect of Photobiomodulation Therapy in the 1500 m Run: An Analysis of Performance and Individual Responsiveness. *Photobiomodul. Photomed. Laser Surg.* **2020**, *38*, 734–742. doi:10.1089/photob.2019.4785.	Randomized, crossover, double-blind placebo-controlled trial. 19 recreationally trained runners.	Mixed wavelength device Cluster: 5 LASERs of 850 nm, 12 LEDs of 670 nm, 8 LEDs of 880 nm, 8 LEDs of 950 nm. Continuous mode (CW).	Before time trial run.	30 J per site, with a total energy dose of 840 J. Exposure duration: 32 s.	1500 m run	Total points irradiated = 28. 14 sites per each lower limb: 8 sites on the quadriceps, 4 sites on the hamstrings, and 2 sites on the gastrocnemius.	Maximum oxygen uptake (VO_2MAX_); Velocity associated with VO_2MAX_ (vVO_2MAX_); PV; Maximal heart rate (HR_MAX_); Respiratory compensa tion point (RCP).	PBMT applied immediately before running in non-controlled environment was not able to improve the 1500 m performance of recreationally trained runners.
[71]	Abreu, J.S.S.; Dos Santos, G.V.; Fonsati, L.; et al. Time-Response of Photobiomodulation Therapy by Light-Emitting Diodes on Muscle Torque and Fatigue Resistance in Young Men: Randomized, Double-Blind, Crossover and Placebo-Controlled Study. *Photobiomodul. Photomed. Laser Surg.* **2020**, *38*, 12, 750–757. doi:10.1089/photob.2020.4813.	Randomized, double-blind, placebo-controlled, crossover trial. 30 healthy and physically active young men, divided into two groups: PBMT (15) and placebo (15).	Flexible array of 132 LEDs: 60 red (635 nm), 72 IR (880 nm). Frequency: 4.7 KHz. Optical power (each LED): 1.2 mW (red) 15 mW (IR). Total optical power: 1152 mW. Area (each LED): 0.2 cm^2^. Effective area of irradiation: 166.75 cm^2^. Power density (each LED): Red: 6 mW/cm^2^; IR 75 mW/cm^2^; irradiation time: 52 s.	5 min, 1 h, 3 h, and 6 h preconditioning PBMT.	Total energy (of the device) 60 J; 1152 mW; 52 s; 166.75 cm^2^, applied on biceps brachii.	Maximal voluntary isometric contractions (MVIC) of elbow flexion.	Biceps brachii (small muscle) = elbow flexor muscles.	Peak torque (PT), rate of torque development (RTD), fatigue resistance, subjective perception of effort in maximal voluntary isometric contractions (MVIC) of elbow flexion.	PBMT was not effective to increase muscle performance and decrease fatigue to demonstrate the possible time–response in humans.

**Table 3:** Effects of experimental PBM on animals.

No	References/Year	Experimental Model	Type of Light/Devices	PBM Before/After Activity	The Fluence Expressed in J/cm^2^	Power Density mW/cm^2^	Parameters Analyzed	Effects	Results
[78]	Macedo, M.M.; Mafra, F.F.P.; Teixeira, C.B.; et al. Photobiomodulation therapy modulates muscle gene ex-pression and improves performance of rats subjected to a chronic resistance exercise protocol. *Photobiomodul. Photomed. Laser Surg.* **2020**, *38*, 12, 713–719. doi:10.1089/photob.2019.4792.	Male Wistar rats in vivo protocols. PBM were applied in direct contact with the skin without shaving on the gastrocnemius anatomical region, 50 s in each paw, 5 days on week for 4 weeks and 9 J per paw totaling 18 J of energy per day.	904 nm GaAs Pulsed 60 mW Number of diodes = 3.	The animals were irradiated before exercise on the hind legs.	6 J/cm^2^ Energy/point 3 J Total energy 18 J Time 50 s each side.	0.12 W/cm^2^	RNA ribosomal 18 s mTOR; myHC; AR; LDH	No statistical difference was observed for Gastrocnemius muscle mass. PBM increased expression of LDH enzyme and gene expression compared to nonirradiated animals.	PBMT did not increase gastrocnemius muscle mass, but improved performance in endurance training, increased expression of performance-related genes and the process of muscle protein synthesis.
[79]	Malta, E.S.; Ferraresi, C.; Monte, M.G.; et al. Effect of 12 weeks of endurance training combined with creatine supplement, photobiomodulation therapy, or both on performance and muscle damage in rats. *Photobiomodul. Photomed. Laser Surg.* **2020**, *38*, 12, 708–712. doi:10.1089/photob.2019.4793.	Twenty-five male Wistar rats weighing similar to 300 g were randomly distributed into five groups.	PBMT was delivered in six points with a laser device 808 nm, 100 mW, 30 s.	After	Energy per point of irradiation: 3 J; 75 J/cm^2^.	100 mW	Peak force and time of force decay during an electrical stimulation protocol CK levels.	PBMT with or without Cr supplement significantly improved performance than all the other groups.	PBMT alone or in conjunction with Cr supplement during a 12-week training program resulted in significantly better muscle performance and lower levels of CK.
[80]	Yamada, E.F.; Bobinski, F.; Martins, D.F.; et al. Photobiomodulation therapy in knee osteoarthritis reduces oxidative stress and inflammatory cytokines in rats. *J. Biophotonics* **2020**, *13*, 1, 201900204. doi:10.1002/jbio.201900204.	Knee osteoarthritis (OA) of the rat induced by monosodium iodoacetate (MIA).	GaAs (gallium-arsenide) 904 nm Pulsed + 9500 Hz.	After	Eight sessions of PBM 3 days/week, 6 or 18 J/cm^2^.	40 mW average radiant power; 70 W of peak radiant power; and 0.1309 cm^2^ spot area.	The inflammatory process, pain and cytokine levels (IL1-β, IL-6, IL-10, TNF-alfa), mechanical and cold hyperalgesia spontaneous pain.	18 J/cm^2^ dose of PBM reduced the pain and polymorpho-nuclear activity of neutrophils in the joint fluid, improved the parameters of oxidative stress in blood serum and spinal cord samples.	PBM improved antioxidant capacity, decreased the level of proinflammatory cytokines, reduced inflammation of the knee joint and pain.
[81]	Neves, L.M.S.; Gonçalves, E.C.D.; Cavalli, J.; et al. Photobiomodulation therapy improves acute inflammatory response in mice: the role of cannabinoid receptors/ATP-sensitive K+ channel/p38-MAPK signalling path-way. *Mol. Neurobiol.* **2018**, *55*, 5580–5593. https://doi.org/10.1007/s12035-017-0792-z.	Male Swiss mice were randomized.	660 nm; AlGaInP (aluminum/gallium/indium/phosphorus) Power: 30 mW, beam area 0.06 cm^2^, and CW output.	After	1, 20, 50, 100, 150, 200 J/cm^2^.	30 mW/cm^2^.	IL-6 and IL-10 catalepsy and motor activity Assessment Involvement of ATP-Sensitive K^+^ Channel and p38 MAP-kinase pathway Thermal nociceptive response test.	PBM (50 J/cm^2^, plantar irradiation) significantly inhibited carrageenan-induced paw oedema through modulation to both CB1 and CB2 cannabinoid receptors. PBMT significantly reduced the level of the pro-inflammatory cytokine IL-6 in both the paws and the spinal cord, as well as the low level of the anti-inflammatory cytokine IL-10 in the spinal cord after carrageenan injection.	PBM opens opportunity for non-pharmacological and non-psychotropic therapy during immune-mediated inflammatory diseases, including ankylosing spondylitis, type 1 diabetes, rheumatoid arthritis, and multiple sclerosis.
[82]	Dos Santos, L.S.; Saltorato, J.C.; Monte, M.G.; et al. PBMT and topical diclofenac as single and combined treatment on skeletal muscle injury in diabetic rats: Effects on biochemical and functional aspects. *Lasers Med. Sci.* **2019**, *34*, 2, 255–262. doi:10.1007/s10103-018-2580-z.	Randomly experimental model of muscle injury through controlled trauma in diabetic rats.	830 nm CW; 0.028 cm^2^ spot area. 3.57 W/cm^2^.		107.51 J/cm^2^ energy density; and 3 J (30 s) dose of energy per point.	100 mW/cm^2^.	Gene expression levels of COX-1 in the anterior tibial muscle. Levels of PGE_2_ in blood samples. Functional index.	PBMT was effective in reducing inflammatory markers (COX-2) and greatly improved the repair process of injured musculoskeletal tissue, at an energy density of 107.1 J/cm^2^ and total energy of 3 J.	PBMT, alone or in combination with diclofenac, decreased the concentration of inflammatory markers and improved the gait of diabetic rats in the acute phase of muscle injury.
[83]	Tomazoni, S.S.; Frigo, L.; dos Reis Ferreira, T.C.; et al. Effects of photobiomodulation therapy and topical non-steroidal anti-inflammatory drug on skeletal muscle injury induced by contusion in rats—Part 1: morphological and functional aspects. *Lasers Med. Sci.* **2017**, *32*, 9, 2111–2120. https://doi.org/10.1007/s10103-017-2346-z.	Ninety-six male Wistar rats were randomized and divided into experimental groups of six animals per group. The animals were submitted to the muscle contusion model produced on the anterior tibial muscle.	830 nm CW; spot area of 0.028 cm^2^, 100 mW.	PBMT was performed one hour after the induction of muscle injury by contusion.	Doses: 35.7; 107.1; 321.4 J/cm^2^ Irradiation time per site (s): 10, 30, 90, as follows: 1 J-35.7 J/cm^2^, 3 J-107.1 J/cm^2^, 9 J-321.4 J/cm^2^. 10, 30, and 90 s.	3.57 W/cm^2^	Morphological analysis—histology. Muscular work Muscle fatigue, tetanic and muscular contraction.	At 6 h, 12 h, and especially 24 h after injury, the three groups treated with PBMT (the best dose of 9 J in total 321.4 J/cm^2^) greatly improved the morphological aspects (organization of muscle fibers and cell nuclei) and inflammation compared to diclofenac group.	PBMT with 3 J (107.1 J/cm^2^), was the most valuable dose of the three used in the study and superior to local NSAID therapy to improve morphological and functional alterations due to muscle injury from contusion.
[84]	Tomazoni, S.S.; Frigo, L.; Dos Reis Ferreira, T.C.; et al. Effects of photobiomodulation therapy and topical non-steroidal anti-inflammatory drug on skeletal muscle injury induced by contusion in rats—Part 2: bio-chemical aspects. *Lasers Med. Sci.* **2017**, *32*, 8, 1879–1887. doi:10.1007/s10103-017-2299-2.	Ninety-six male Wistar rats were randomized. Muscle injury was induced by trauma to the anterior tibial muscle of rats.	830 nm CW; spot area of 0.028 cm^2^, 100 mW	PBMT was performed one hour after the induction of muscle injury by contusion.	Doses 35.7; 107.1; 321.4 J/cm^2^ Irradiation time per site (s) 10, 30, 90.	3.57 W/cm^2^	Gene expression of TNF- α and COX-2 (for 6, 12 and 24 h) in the anterior tibialis muscle ELISA for the detection of TNF-α IL-1β and IL-6 expressions and β-actin. PBMT activates the biostimulation process, which accelerates the resolution of acute inflammatory response and tissue regeneration.	PBMT 1 J (35.7 J/ cm^2^), 3 J (107.1 J/cm^2^), and 9 J (321.4 J/cm^2^) reduced levels of TNF-α, IL-1β, and IL-6 at all assessed times as compared to the injury and diclofenac groups.	PBMT with 3 J (107.1 J/cm^2^), was the most valuable dose of the three used in the study and superior to local NSAID therapy for improvement after muscle injuries from contusion. PBMT may be suggested as the best alternative for the treatment of muscle contusion given its role in the modulation of inflammation and consequently in tissue repair.
[85]	Silva, G.; Ferraresi, C.; de Almeida, R.T.; et al. Insulin resistance is improved in high-fat fed mice by photo-biomodulation therapy at 630 nm. *J. Biophotonics* **2020**, *13*, 3, e201960140. doi:10.1002/jbio.201960140.	Thirty-four 8-week-old male Swiss albino mice.	630 ± 20 nm CW.	Before and after	31.188 J/cm^2^ Energy delivered per site: 12 J. No. of irradiation sites: 5. Total irradiation time 200 s. Total energy delivered per day: 60 J.	779.53 mW/cm^2^	Histological analysis Protein analysis Blood glucose concentration Effect of low-fat diet (LFD) or high-fat diet (HFD). Adipocyte hypertrophy and inflammatory infiltrate.	PBMT improved glucose tolerance, insulin resistance and fasting hyperinsulinemia.	PBMT at 630 nm, CW, improved insulin resistance and glucose metabolism in HFD-fed mice.
[86]	De Brito Vieira, W.H.; Ferraresi, C.; Schwantes, M.L.B.; et al. Photobiomodulation increases mitochondrial citrate synthase activity in rats submitted to aerobic training. *Lasers Med. Sci.* **2018**, *33*, 4, 803–810. https://doi.org/10.1007/s10103-017-2424-2.	Fifty-four rats were allocated into four groups.	780 nm (GaAlAs). Beam area of 0.04 cm^2^.Energy per point of 0.15 J. Total energy of 1.2 J (per session for animal). Time of irradiation per point equal 10 s; CW; during 30 days of training.	After physical effort (days of training and effort tests).	Fluency of 3.8 J/cm^2^.	Irradiance of 37.5 mW/cm^2^.	LDH and CS activity LDH/CS ratios.	CS activity in the heart and soleus muscles in the exercise and PBM group was significantly higher, and LDH activity was lower (soleus muscle) than in the other groups.	PBM and treadmill aerobic training together participate in increasing the oxidative capacity especially of tissues with aerobic metabolism, such as in the soleus and heart muscles.
[87]	Frigero, M.; dos Santos, S.A.; Serra, A.J.; et al. Effect of photobiomodulation therapy on oxidative stress markers of gastrocnemius muscle of diabetic rats subjected to high-intensity exercise. *Lasers Med. Sci.* **2018**, *33*, 8, 1781–1790. https://doi.org/10.1007/s10103-018-2540-7.	Twenty-four male Wistar diabetic rats subjected to high-intensity exercise, were randomly allocated to groups of eight animals each; this study comprised 16 diabetic (with fatigue, and PBMT diabetic fatigue) and eight control rats.	808 nm 30 mW 0.028 cm^2^	Prior to each training session.	Total energy (Joule) = 24 142.4 J/cm^2^	1.071 mW/cm^2^	Blood lactate concentrations High-intensity exercise Volumes of oxygen (VO_2_) and carbon dioxide (VCO_2_). VO_2_ma xConcentrations of TBARS/MDA SOD, CAT, GPx activity GPx and SOD.	The PBMT diabetic fatigue group was irradiated before starting the exercises, with a dose of 4 J and 808 nm, and were subjected to running with speed and gradually slope to exhaustion. Analyzes of CAT, SOD and GPx activities were found to be significantly higher in the PBMT fatigue group than in the diabetic fatigue group.	PBM can reduce oxidative stress and may be an alternative treatment option to support fitness when subjects are unable to activate.
[88]	Da Silva Neto Trajano, L.A.; Trajano, E.T.L.; da Silva Sergio, L.P.; et al. Photobiomodulation effects on mRNA levels from genomic and chromosome stabilization genes in injured muscle. *Lasers Med. Sci.* **2018**, *33*, 7, 1513–1519. https://doi.org/10.1007/s10103-018-2510-0.	Cryoinjury was induced by two applications of a metal probe cooled in liquid nitrogen directly on the tibialis anterior muscle in rat. Wistar male rats were randomly divided into six groups.	904 nm GaAs	after injury.	3 J/cm^2^ 3 J/cm^2^ Four irradiation procedures (3 J/cm^2^ per irradiation, totaling 12 J/cm^2^.	25 mW 75 mW	Total mRNA extraction Complementary DNA (cDNA) synthesis Telomeric repeat binding factors (TRF1 and TRF2), ATM serine/threonine kinase. Tumor protein p53 (P53), Glyceraldehyde-3- phosphate dehydrogenase (GAPDH) primers.	PBM (904 nm, 3 J/cm^2^ per irradiation, 25 and 75 mW, 4 irradiations) significantly reduced TRF1 mRNA levels in injured muscle exposed to laser, compared to the injury group.	PBM at 25 and 75 mW reduced the mRNA levels from ATM and p53, as well as mRNA levels from TRF1 and TRF2 in injured skeletal muscle. In conclusion, PBM altered mRNA relative levels from the genes related to genomic and telomere stabilization in injured skeletal muscle.
[89]	Ferraresi, C.; de Sousa, M.V.; Huang, Y.Y.; et al. Time response of increases in ATP and muscle resistance to fatigue after low-level laser (light) therapy (LLLT) in mice. *Lasers Med. Sci.* **2015**, *30*, 1259–1267. doi:10.1007/s10103-015-1723-8.	Fifty male Balb/c mice were randomly allocated into two equal groups: LEDT-ATP and LEDT-fatigue. Both groups were subdivided into five equal subgroups: LEDT-sham, LEDT-5 min, LEDT-3 h, LEDT-6 h, and LEDT-24 h.	Forty LEDs: 20 red (630 ± 10 nm, 25 mW); 20 infrared (850 ± 20 nm, 50 mW). CW.	LEDT applied to legs, gluteus, and lower back muscles, as follows: LEDT-sham; LEDT-5 min before; LEDT-3 h before; LEDT-6 h before; LEDT-24 h before.	Optical output each LED: 50 mW (IR) and 25 mW (RED). Optical output (cluster): 1000 mW (IR) and 500 mW (RED). Treatment time: 90 s Energy density: 7.2 J/cm^2^.	Power density: 80 mW/cm^2^. Distance from mice or power meter: 45 mm.	Fatigue test was performed by mice repeatedly climbing an inclined ladder bearing a load of 150% of body weight until exhaustion.	Time response for LEDT-mediated increase in adenosine triphosphate (ATP) in the soleus and gastrocnemius muscles. LEDT effects on the resistance of muscles to fatigue during intense exercise.	LEDT-6 h had the highest muscle ATP content and the highest number of repetitions in the fatigue test, compared to all subgroups. Conclusion: LEDT increased ATP content in muscles and fatigue resistance in mice with a peak at 6 h.
[90]	Ferraresi, C.; Parizotto, N.A.; Pires de Sousa, M.V.; et al. Light-emitting diode therapy in exercise-trained mice increases muscle performance, cytochrome c oxidase activity, ATP and cell proliferation [J. Biophotonics 8, No. 9, 740–754 (2015)]. *J. Biophotonics* **2016**, *9*, 976. doi:10.1002/jbio.201680087.	Twenty-two male Balb/c mice were randomly divided into 5 groups: LEDT-Sham group (5); LEDT-Before (5); LEDT-Before-After (5); LEDT-After (5); Control (2): no LEDT, no exercise. LEDT over both legs, gluteus and lower-back muscles at a distance of 45 mm (without contact).	Forty LEDs (20 red –630 ± 10 nm; 20 infrared −850 ± 20 nm) with diameter of 76 mm. CW.	Complex protocol depending on group: - Sham; - Before; - Before and after; - After; control no LEDT, no exercise.	Optical output each LED: 50 mW (IR) and 25 mW (RED) optical output (cluster): 1000 mW (IR) and 500 mW (RED). Treatment time: 90 s. Energy density applied (at skin surface): 7.2 J/cm^2^.	LED cluster size: 45 cm^2^. Power density (at skin surface): 80 mW/cm^2^. Application mode: without contact. Distance from mice or power meter: 45 mm.	Three repetitions maximum load (3 RM) After 24 h from initial 3 RM baseline: 6 training sessions on alternate days (every 48 h), as follows: 5 sets of 10 repetitions (climbs) on the ladder with a rest period of 2 min between each set. - Distance climbed (in cm); - number of repetitions/set; - time/exercise. From these data were calculated: - muscle work and - muscle power in each training session.	Evaluation of the muscle performance in each group; muscular ATP; muscular glycogen; Oxidative stress markers; Immunofluorescence analyses Cytochrome c oxidase subunit IV.	Clear improvement in muscle performance, energy metabolism, oxidative stress defense and repair/proliferation with different regimens of LEDT applied to muscles in conjunction with a training regimen. Six bi-daily training sessions LEDT-After and LEDT-Before-After regimens more than doubled muscle performance and increased ATP more than tenfold.
[91]	Tomazoni, S.S.; Leal-Junior, E.C.; Frigo, L.; et al. Isolated and combined effects of photobiomodulation therapy, topical nonsteroidal anti-inflammatory drugs, and physical activity in the treatment of osteoarthritis induced by papain. *J. Biomed. Opt.* **2016**, *21*, 10, 108001. doi:10.1117/1.JBO.21.10.108001.	Fifty-four Wistar rats were randomly divided into experimental groups: NSAID, physical activity, and PBMT applied alone and/or in combination between groups.	830 nm CW, spot area of 0.028 cm^2^, 100 mW.	Twenty-one days after the last injection of papain to induce OA, PBM was applied 3 times a week (every other day) for 8 consecutive weeks for a total of 24 therapy sessions.	214; 2 J∕cm^2^, 6 J per point, 60 s per point, 1 point on the OA join, in direct contact with the skin.	35.71 W/cm^2^.	Histologic characterization of the knee joint. The total amount of cells in the articular cavity. MPO; HPRT; MMPs activity. Gene Expression of MMP-3 and MMP-13 by RT-PCR.	PBMT was the most effective for improving the parameters investigated, had no negative side effects or restrictions in musculoskeletal disorders.	PBMT was the best alternative among the therapies tested in this study because it has improved a lot of morphological changes and enzymes that were involved in joint damage.
[92]	Yang, L.; Dong, Y.; Wu, C.; et al. Photobiomodulation preconditioning prevents cognitive impairment in a neonatal rat model of hypoxia-ischemia. *J. Biophotonics* **2019**, *12*, 6, e201800359. https://doi.org/10.1002/jbio.201800359.	Ten-day-old neonatal Sprague-Dawley rats.	808 nm, CW.	PBM preconditioning.	12 J/cm^2^.	100 mW/cm^2^.	ATP contents in total proteins. Tests at P28 and P29 were conducted to test the recognition memory. The shrinkage volume and neuronal density in the hippocampal CA1 region HI-induced changes in mitochondrial fragmentation in hippocampal CA1 region on P16. Content of cytochrome c in mitochondria and cytosol, the activities of caspase-9 and caspase-3, and apoptosis after HI insults.	PBM can turn off the release of cytochrome C from mitochondria to cytoplasm followed by arresting mitochondria-mediated apoptotic pathway and neuronal apoptosis.	Authors concluded that PBM pre-treatment could suppress mitochondria-mediated apoptotic pathway and neuronal apoptosis by the preservation of mitochondria in an HI model. PBM to human infants who have already suffered an HI insult could improve the prognosis of this condition.
[93]	De Oliveira, H.A.; Antonio, E.L.; Silva, F.A.; et al. Protective effects of photobiomodulation against resistance exercise-induced muscle damage and inflammation in rats. *J. Sports Sci.* **2018**, *36*, 20, 2349–2357. doi:10.1080/02640414.2018.1457419.	Female Wistar rats were randomized.	830 nm spot area of 0.028 cm^2^, 100 mW.	Prior to (upper panel) and post-exercise	71.4 J/cm^2^ 142.8 J/cm^2^ 285.6 J/cm^2^ Energy per point (J): 2, 4, 8.	3.57 W/cm^2^	The blood levels of lactate, CK, and LDH Gene expression and skeletal muscle inflammation makers: TNF-α, IL-6, IL1-β, IL-10. Skeletal muscle macrophage infiltration: CD68, CINC-1, MCP-1. Morphological analysis—histology immunohistochemical assays. Familiarization and dynamic resistance exercise.	PBM reduced muscles damage induced by resistance exercise and decreased the CK and LDH levels at 4 J dose. PBM after exercise decreased muscle levels of IL-6, IL-1β, CINC-1, MCP-1 and IL-10 more than those in the control group at 24 h post-exercises. Decrease in CINC-1 may suggest a reduction in muscle oxidation due to growth of antioxidant activity.	PBM administered before and after exercise at a dose of 4 J reduces muscle destruction and inflammation. PBM could protect athletes from muscle injuries during exercise and accelerates repairs when they occur.

## Data Availability

The original data and references used to support the findings of this study are available from the first and corresponding author upon request.

## Abbreviations

AP	Aerobic performance
AR	Active recovery
AR	Androgen receptor
AT	Anaerobic threshold
ATM	Ataxia telangiectasia mutated
ATP	Adenosine triphosphate
CAT	Catalase
CB1 and CB2	Cannabinoid receptors 1 and 2
CBF	Cerebral blood flow
CCO	Cytochrome c oxidase
cDNA	Complementary DNA
CINC-1	Cytokine-induced neutrophil chemoattractant-1
CK	Creatine kinase
CMJ	Counter movement jump
COD	Crossover design
CON	Concentric
COX-1/COX-2	Cyclooxygenases- 1 and -2
Cr	Creatine
CS	Citrate synthase
CSA	Larger cross-sectional area
CW	Continuous wave
CWI	Cold water immersion
DOMS	Delayed onset muscle soreness
ECC	Eccentric
GAPDH	Glyceraldehyde-3-phosphate dehydrogenase primers
GPCRs	G-protein-coupled receptors
GPx	Glutathione peroxidase
HFD	High-fat diet
HI	Neonatal hypoxia-ischemia
HPRT	Hypoxanthine-guanine phosphoribosyl transferase
HR	Heart rate
HRV	Heart rate variability
HSI	Hamstring strain injury
ICC	Intra-class correlation coefficient
IL-10	Interleukin 10
IL-1β	Interleukin-1β
IL-6	Interleukin-6
IMP	Impulse
IR	Infrared
iVO2_MAX_	Intensity at which VO2_MAX_ was reached
KET test	Knee extension test
La−P	Peak lactate concentration after exercise
LDH	Lactate dehydrogenase
LEDs	Light-emitting diodes
LEDT	Light-emitting diode therapy
LFD	Low-fat diet
MAOD	Maximal accumulated oxygen deficit
MAODALT	Alternative maximal accumulated oxygen deficit
MAP-2	Microtubule-associated protein-2
MCP-1	Monocyte chemoattractant protein-1
MDA	Level of malondialdehyde
MHFAKE	Maximal hip flexion active knee extension
MMPs	Matrix metalloproteinases
MOP	Mean output power
MPO	Myeloperoxidase
MRF4	Myogenic regulatory factor (herculin)
MRS	Maximum running speed
mT	mili Testa (magnetic field)
mTOR	Mammalian (or mechanistic) target of rapamycin
MVC	Maximum voluntary contraction
myHC	Myosin heavy chain
NMES	Neuromuscular electrical stimulation
Nrf2	Nuclear factor erythroid 2-related factor 2
NSAIDs	Non-steroidal anti-inflammatory drugs
OA	Osteoarthritis
P200	Performance of 200 m
P53	Tumor protein p53
PBMT	Photobiomodulation therapy
PCR	Polymerase chain reaction
PF	Peak force
PGE2	Prostaglandin E2
PNS	Peripheral nerve stimulation
PPO	Peak power output
PSLR	Passive straight leg raise
PV	Peak velocity
R	Red
RCT	Double-blind randomized controlled trial
RER_EX_	Exhaustion respiratory exchange ratio
RCTs	Placebo-controlled randomized trials
RPE	Rating of perceived exertion
RPEex	Exhaustion perceived exertion
SIT	Sprint interval training
SJ	Squat jump
SLR	Straight leg raise
sMF	Static magnetic field
SmO_2_	Muscle oxygen saturation
SOD	Superoxide dismutase
SSC	Stretch-shortening cycle
TAC	Total antioxidant capacity
TBARS	2-thiobarbituric acid
TNF-α	Tumor necrosis factor-alpha
TRF1/TRF2	Telomeric repeat binding factors
VCO2	Volume carbon dioxide
VO2	O2 uptake
VO2_EX_	Exhaustion oxygen consumption
VO2_MAX_	Maximal oxygen uptake
YYIR1	YoYo intermittent recovery level 1
1-RM	Load in 1-repetition maximum test
30 CJ	30-s crossbar jump test
↑	Increase
↓	Decrease
